# Reliable and efficient a posteriori error estimation for adaptive IGA boundary element methods for weakly-singular integral equations

**DOI:** 10.1016/j.cma.2015.03.013

**Published:** 2015-06-15

**Authors:** Michael Feischl, Gregor Gantner, Dirk Praetorius

**Affiliations:** Vienna University of Technology, Institute for Analysis and Scientific Computing, Wiedner Hauptstraße 8-10, A-1040 Wien, Austria

**Keywords:** Isogeometric analysis, Boundary element method, A posteriori error estimate, Adaptive mesh-refinement

## Abstract

We consider the Galerkin boundary element method (BEM) for weakly-singular integral equations of the first-kind in 2D. We analyze some residual-type a posteriori error estimator which provides a lower as well as an upper bound for the unknown Galerkin BEM error. The required assumptions are weak and allow for piecewise smooth parametrizations of the boundary, local mesh-refinement, and related standard piecewise polynomials as well as NURBS. In particular, our analysis gives a first contribution to adaptive BEM in the frame of isogeometric analysis (IGABEM), for which we formulate an adaptive algorithm which steers the local mesh-refinement and the multiplicity of the knots. Numerical experiments underline the theoretical findings and show that the proposed adaptive strategy leads to optimal convergence.

## 1 Introduction

**Isogeometric analysis.** The central idea of isogeometric analysis is to use the same ansatz functions for the discretization of the partial differential equation at hand, as are used for the representation of the problem geometry. Usually, the problem geometry Ω is represented in computer aided design (CAD) by means of NURBS or T-splines. This concept, originally invented in  [1] for finite element methods (IGAFEM) has proved very fruitful in applications  [1,2]; see also the monograph  [3]. Since CAD directly provides a parametrization of the boundary ∂Ω, this makes the boundary element method (BEM) the most attractive numerical scheme, if applicable (i.e., provided that the fundamental solution of the differential operator is explicitly known). Isogeometric BEM (IGABEM) has first been considered for 2D BEM in  [4] and for 3D BEM in  [5]. Unlike standard BEM with piecewise polynomials which is well-studied in the literature, cf. the monographs  [6,7] and the references therein, the numerical analysis of IGABEM is essentially open. We only refer to  [2,8–10] for numerical experiments and to  [11] for some quadrature analysis. In particular, a posteriori error estimation has been well-studied for standard BEM, e.g.,  [12–18] as well as the recent overview article  [19], but has not been treated for IGABEM so far. The purpose of the present work is to shed some first light on a posteriori error analysis for IGABEM which provides some mathematical foundation of a corresponding adaptive algorithm.

**Main result.** Let $\Omega \subset R^{2}$ be a Lipschitz domain and Γ⊆∂Ω be a compact, piecewise smooth part of the boundary with finitely many connected components (see Sections  2.2 and 2.3). Given a right-hand side f, we consider boundary integral equations in the abstract form

(1.1) $$V\phi \left(x\right)=f\left(x\right)\;\text{for all}\;x\in \Gamma_{}\text{,}$$

where $V:{\overset{˜}{H}}^{-1/2}\left(\Gamma_{}\right)\to H^{1/2}\left(\Gamma_{}\right)$ is an elliptic isomorphism. Here $H^{1/2}\left(\Gamma_{}\right)$ is a fractional-order Sobolev space, and ${\overset{˜}{H}}^{-1/2}\left(\Gamma_{}\right)$ is its dual (see Section  2). Given $f\in H^{1/2}\left(\Gamma_{}\right)$, the Lax–Milgram lemma provides existence and uniqueness of the solution $\phi \in {\overset{˜}{H}}^{-1/2}\left(\Gamma_{}\right)$ of the variational formulation of  (1.1)

(1.2) $$\int_{\Gamma_{}}V\phi \left(x\right)\psi \left(x\right)\;dx=\int_{\Gamma_{}}f\left(x\right)\psi \left(x\right)\;dx\;\text{for all}\;\psi \in {\overset{˜}{H}}^{-1/2}\left(\Gamma_{}\right)\text{.}$$

In the Galerkin boundary element method (BEM), the test space ${\overset{˜}{H}}^{-1/2}\left(\Gamma_{}\right)$ is replaced by some discrete subspace $X_{h}\subseteq L^{2}\left(\Gamma_{}\right)\subseteq {\overset{˜}{H}}^{-1/2}\left(\Gamma_{}\right)$. Again, the Lax–Milgram lemma guarantees existence and uniqueness of the solution $\phi_{h}\in X_{h}$ of the discrete variational formulation

(1.3) $$\int_{\Gamma_{}}V\phi_{h}\left(x\right)\psi_{h}\left(x\right)\;dx=\int_{\Gamma_{}}f\left(x\right)\psi_{h}\left(x\right)\;dx\;\text{for all}\;\psi_{h}\in X_{h}\text{,}$$

and $\phi_{h}$ can in fact be computed by solving a linear system of equations.

We assume that $X_{h}$ is linked with a partition $T_{h}$ of Γ into a set of connected segments. For each vertex $z\in N_{h}$ of $T_{h}$, let $\omega_{h}\left(z\right)≔⋃\left\{T\in T_{h}\;:\;z\in T\right\}$ denote the node patch. If $X_{h}$ is sufficiently rich (e.g.,  $X_{h}$ contains certain splines or NURBS; see Section  4), we prove that

(1.4) $$C_{\mathrm{rel}}^{-1}\;{\left\|\phi -\phi_{h}\right\|}_{{\overset{˜}{H}}^{-1/2}\left(\Gamma_{}\right)}\leq \eta_{h}≔{\left(\underset{z\in N_{h}}{\sum }\overset{2}{\underset{H^{1/2}\left(\omega_{h}\left(z\right)\right)}{\left|r_{h}\right|}}\right)}^{1/2}\leq C_{\mathrm{eff}}\;{\left\|\phi -\phi_{h}\right\|}_{{\overset{˜}{H}}^{-1/2}\left(\Gamma_{}\right)}$$

with some $X_{h}$-independent constants $C_{\mathrm{eff}},C_{\mathrm{rel}}>0$, i.e., the unknown BEM error is controlled by some computable a posteriori error estimator $\eta_{h}$. Here, $r_{h}≔f-V\phi_{h}\in H^{1/2}\left(\Gamma_{}\right)$ denotes the residual and

(1.5) $${\left|r_{h}\right|}_{H^{1/2}\left(\omega_{h}\left(z\right)\right)}≔\int_{\omega_{h}\left(z\right)}\int_{\omega_{h}\left(z\right)}\frac{{\left|r_{h}\left(x\right)-r_{h}\left(y\right)\right|}^{2}}{{\left|x-y\right|}^{2}}\;dy\;dx$$

is the Sobolev–Slobodeckij seminorm.

Estimate  (1.4) has first been proved by Faermann  [17] for closed $\Gamma_{}=\partial \Omega$ and standard spline spaces $X_{h}$ based on the arclength parametrization γ:[0,L]→Γ. In isogeometric analysis, γ is *not* the arclength parametrization. In our contribution, we generalize and refine the original analysis of Faermann  [17]: Our analysis allows, first, closed as well as open parts of the boundary, second, general piecewise smooth parametrizations γ and, third, covers standard piecewise polynomials as well as NURBS spaces $X_{h}$.

**Outline.** Section  2 recalls the functional analytic framework, provides the assumptions on Γ and its parametrization γ, and fixes the necessary notation. The proof of  (1.4) is given in Section  3 for sufficiently rich spaces $X_{h}$ (Theorem 3.1). In Section  4, we recall the NURBS spaces for IGABEM and prove that these spaces $X_{h}$ satisfy the assumptions (Assumptions (A1)–(A2) in Section  3.1) of the a posteriori error estimate  (1.4). Based on knot insertion, we formulate an adaptive algorithm which is capable to control and adapt the multiplicity of the nodes as well as the local mesh-size (Algorithm 4.5). Section  5 gives some brief comments on the stable implementation of adaptive IGABEM for Symm’s integral equation and provides the numerical evidence for the superiority of the proposed adaptive IGABEM over IGABEM with uniform mesh-refinement. In the final Section  6, some conclusions are drawn and some comments on future work and open questions are reported.

## 2 Preliminaries

The purpose of this section is to collect the main assumptions on the boundary and its discretization as well as to fix the notation. For more details on Sobolev spaces and the functional analytic setting of weakly-singular integral equations, we refer to the literature, e.g., the monographs  [20,21,6] and the references therein.

Throughout, |⋅| denotes the absolute value of scalars, the Euclidean norm of vectors in $R^{2}$, the measure of a set in R, e.g. the length of an interval, or the arclength of a curve in $R^{2}$. The respective meaning will be clear from the context.

### 2.1 Sobolev spaces

For any measurable subset ω⊆Γ, let $L^{2}\left(\omega \right)$ denote the Lebesgue space of all square integrable functions which is associated with the norm ${\left\|u\right\|}_{L^{2}\left(\omega \right)}^{2}≔\int_{\omega }{\left|u\left(x\right)\right|}^{2}\;dx$. We define the Hilbert space

(2.1) $$H^{1/2}\left(\omega \right)≔\left\{u\in L^{2}\left(\omega \right)\;:\;{\left\|u\right\|}_{H^{1/2}\left(\omega \right)}<\infty \right\}\text{,}$$

associated with the Sobolev–Slobodeckij norm

(2.2) $${\left\|u\right\|}_{H^{1/2}\left(\omega \right)}^{2}≔{\left\|u\right\|}_{L^{2}\left(\omega \right)}^{2}+{\left|u\right|}_{H^{1/2}\left(\omega \right)}^{2}\;\text{with}\;{\left|u\right|}_{H^{1/2}\left(\omega \right)}^{2}≔\int_{\omega }\int_{\omega }\frac{{\left|u\left(x\right)-u\left(y\right)\right|}^{2}}{{\left|x-y\right|}^{2}}\;dy\;dx\text{.}$$

For finite intervals I⊆R we use analogous definitions. By ${\overset{˜}{H}}^{-1/2}\left(\omega \right)$, we denote the dual space of $H^{1/2}\left(\omega \right)$, where duality is understood with respect to the $L^{2}\left(\omega \right)$-scalar product, i.e., 

(2.3) $$\langle u\;;\;\phi \rangle =\int_{\omega }u\left(x\right)\phi \left(x\right)\;dx\;\text{for all}\;u\in H^{1/2}\left(\omega \right)\;\text{and}\;\phi \in L^{2}\left(\omega \right)\text{.}$$

We note that $H^{1/2}\left(\Gamma \right)\subseteq L^{2}\left(\Gamma \right)\subseteq {\overset{˜}{H}}^{-1/2}\left(\Gamma \right)$ form a Gelfand triple and all inclusions are dense and compact. Amongst other equivalent definitions of $H^{1/2}\left(\omega \right)$ are the characterization as trace space of functions in $H^{1}\left(\Omega \right)$ as well as equivalent interpolation techniques. All these definitions provide the same space of functions but different norms, where norm equivalence constants depend only on ω; see, e.g., the monograph  [21] and references therein. Throughout, we shall use the Sobolev–Slobodeckij norm (2.2), since it is numerically computable.

### 2.2 Connectedness of Γ

Let the part of the boundary $\Gamma ={⋃}_{i}\Gamma_{i}$ be decomposed into its finitely many connected components $\Gamma_{i}$. The $\Gamma_{i}$ are compact and piecewise smooth as well. Note that this yields existence of some constant c>0 such that |x−y|≥c>0 for all $x\in \Gamma_{i}$, $y\in \Gamma_{j}$, and i≠j. Together with ${\left|u\left(x\right)-u\left(y\right)\right|}^{2}\leq 2\;{\left|u\left(x\right)\right|}^{2}+2\;{\left|u\left(y\right)\right|}^{2}$, this provides the estimate

∑i,ji≠j∫Γi∫Γj|u(x)−u(y)|2|x−y|2dydx≲∑i‖u‖L2(Γi)2+∑j‖u‖L2(Γj)2≃‖u‖L2(Γ)2

and results in norm equivalence

‖u‖H1/2(Γ)2=∑i‖u‖H1/2(Γi)2+∑i,ji≠j∫Γi∫Γj|u(x)−u(y)|2|x−y|2dydx≃∑i‖u‖H1/2(Γi)2.

The usual piecewise polynomial and NURBS basis functions have connected support and are hence supported by some *single* $\Gamma_{i}$ each. Without loss of generality and for the ease of presentation, we may therefore from now on assume that Γ is connected. All results of this work remain valid for non-connected Γ.

### 2.3 Boundary parametrization

We assume that either Γ=∂Ω is parametrized by a closed continuous and piecewise two times continuously differentiable path γ:[a,b]→Γ such that the restriction $\gamma {|}_{\left[a,b\right)}$ is even bijective, or that Γ⫋∂Ω is parametrized by a bijective continuous and piecewise two times continuously differentiable path γ:[a,b]→Γ. In the first case, we speak of *closed* Γ=∂Ω, whereas the second case is referred to as *open* Γ⫋∂Ω. For closed Γ, we denote the (b−a)-periodic extension to R also by γ. For the left and right derivative of γ, we assume that $\gamma^{\prime_{\ell }}\left(t\right)\neq 0$ for t∈(a,b] and $\gamma^{\prime_{r}}\left(t\right)\neq 0$ for t∈[a,b). Moreover we assume that $\gamma^{\prime_{\ell }}\left(t\right)+c\gamma^{\prime_{r}}\left(t\right)\neq 0$ for all c>0 and t∈[a,b] resp. t∈(a,b). Finally, let $\gamma_{L}:\left[0,L\right]\to \Gamma$ denote the arclength parametrization, i.e.,  $\left|\gamma_{L}^{\prime_{\ell }}\left(t\right)\right|=1=\left|\gamma_{L}^{\prime_{r}}\left(t\right)\right|$, and its periodic extension. Then, elementary differential geometry yields bi-Lipschitz continuity

(2.4) $$C_{\Gamma }^{-1}\leq \frac{\left|\gamma_{L}\left(s\right)-\gamma_{L}\left(t\right)\right|}{\left|s-t\right|}\leq C_{\Gamma }\;\text{for}\;s,t\in R,\;\text{with}\;\left\{ \begin{array}{c} \left|s-t\right|\leq \frac{3}{4}\;L,\text{ for closed }\Gamma \text{,}\\ s\neq t\in \left[0,L\right],\text{ for open }\Gamma \text{.} \end{array}\right.$$

A proof is given in  [22, Lemma 2.1] for closed Γ. For open Γ, the proof is even simpler. If Γ is closed and $\left|I\right|\leq \frac{3}{4}L$ resp. if Γ is open and I⊆[a,b], we see from (2.4) that

(2.5) $$C_{\Gamma }^{-1}{\left|u\circ \gamma_{L}\right|}_{H^{1/2}\left(I\right)}\leq {\left|u\right|}_{H^{1/2}\left(\gamma_{L}\left(I\right)\right)}\leq C_{\Gamma }{\left|u\circ \gamma_{L}\right|}_{H^{1/2}\left(I\right)}\text{.}$$

### 2.4 Boundary discretization

The part of the boundary Γ is split into a set $T_{h}=\left\{T_{1},\ldots ,T_{n}\right\}$ of compact and connected segments $T_{j}$. The endpoints of the elements of $T_{h}$ form the set of nodes $N_{h}≔\left\{z_{j}\;:\;j=1,\ldots ,n\right\}$ for closed Γ and $N_{h}=\left\{z_{j}\;:\;j=0,\ldots ,n\right\}$ for open Γ. The arclength of each element $T\in T_{h}$ is denoted by $h_{T}$, where $h≔\max_{T\in T_{h}}h_{T}$. Moreover, we define the *shape regularity constant*

$$\kappa \left(T_{h}\right)≔\max\left(\left\{h_{T}/h_{T^{\prime }}\;:\;T,T^{\prime }\in T_{h},T\cap T^{\prime }\neq 0̸\right\}\right)\text{.}$$

For closed Γ, we extend the nodes, elements and their length periodically. We suppose

(2.6) h≤|Γ|/4,

if Γ is closed.

### 2.5 Parameter domain discretization

Given the parametrization γ:[a,b]→Γ, the discretization $T_{h}$ induces a discretization ${\overset{ˇ}{T}}_{h}=\left\{{\overset{ˇ}{T}}_{1},\ldots ,{\overset{ˇ}{T}}_{n}\right\}$ on the parameter domain [a,b]. Let $a={\overset{ˇ}{z}}_{0}<{\overset{ˇ}{z}}_{1}<\cdots <{\overset{ˇ}{z}}_{n}$ be the endpoints of the elements of ${\overset{ˇ}{T}}_{h}$. We assume ${\overset{ˇ}{T}}_{j}=\left[{\overset{ˇ}{z}}_{j-1},{\overset{ˇ}{z}}_{j}\right]$, $\gamma \left(\overset{ˇ}{T_{j}}\right)=T_{j}$ and $\gamma \left({\overset{ˇ}{z}}_{j}\right)=x_{j}$. We define ${\overset{ˇ}{N}}_{h}≔\left\{{\overset{ˇ}{z}}_{j}\;:\;j=1,\ldots ,n\right\}$ for closed Γ=∂Ω, and ${\overset{ˇ}{N}}_{h}≔\left\{{\overset{ˇ}{z}}_{j}\;:\;j=0,\ldots ,n\right\}$ for open Γ⫋∂Ω. The length of each element $\overset{ˇ}{T}\in {\overset{ˇ}{T}}_{h}$ is denoted by $h_{\overset{ˇ}{T}}$. Moreover, we define the *shape regularity constant* on [a,b] as

$$\kappa \left(\overset{ˇ}{T_{h}}\right)≔\max\left(\left\{h_{\overset{ˇ}{T}}/h_{{\overset{ˇ}{T}}^{\prime }}\;:\;\overset{ˇ}{T},{\overset{ˇ}{T}}^{\prime }\in \overset{ˇ}{T_{h}},\gamma \left(\overset{ˇ}{T}\right)\cap \gamma \left({\overset{ˇ}{T}}^{\prime }\right)\neq 0̸\right\}\right)\text{.}$$

## 3 A posteriori error estimate

### 3.1 Main theorem

For $T\in T_{h}$, we inductively define the patch $\omega_{h}^{m}\left(T\right)\subseteq \Gamma$ of order $m\in N_{0}$ by

(3.1) $$\omega_{h}^{0}\left(T\right)≔T\text{,}\;\omega_{h}^{m+1}\left(T\right)≔⋃\left\{T^{\prime }\in T_{h}\;:\;T^{\prime }\cap \overset{m}{\underset{h}{\omega }}\left(T\right)\neq 0̸\right\}\text{.}$$

The main result of Theorem 3.1 requires the following two assumptions on $T_{h}$ and $X_{h}$ for some fixed integer $m\in N_{0}$:

(A1) For each $T\in T_{h}$, there exists some fixed function $\psi_{T}\in X_{h}$ with connected support $\mathrm{supp}\left(\psi_{T}\right)$ such that
  (3.2) $$T\subseteq \mathrm{supp}\left(\psi_{T}\right)\subseteq \omega_{h}^{m}\left(T\right)\text{.}$$
(A2) There exists some constant q∈(0,1] such that
  (3.3) $${\left\|1-\psi_{T}\right\|}_{L^{2}\left(\mathrm{supp}\left(\psi_{T}\right)\right)}^{2}\leq \left(1-q\right)\;\left|\mathrm{supp}\left(\psi_{T}\right)\right|\;\text{for all}\;T\in T_{h}\text{.}$$

With these assumptions, we can formulate the following theorem which states validity of  (1.4). For standard BEM and piecewise polynomials based on the arclength parametrization $\gamma_{L}$ of some closed boundary Γ=∂Ω, the analogous result is first proved in  [17, Theorem 3.1]

Theorem 3.1 *The residual* $r_{h}=f-V\phi_{h}$ *satisfies the efficiency estimate*

(3.4) $$\eta_{h}≔{\left(\underset{z\in N_{h}}{\sum }\overset{2}{\underset{H^{1/2}\left(\omega_{h}\left(z\right)\right)}{\left|r_{h}\right|}}\right)}^{1/2}\leq C_{\mathrm{eff}}\;{\left\|\phi -\phi_{h}\right\|}_{{\overset{˜}{H}}^{-1/2}\left(\Gamma_{}\right)}\text{.}$$

*If the mesh* $T_{h}$ *and the discrete space* $X_{h}$ *satisfy assumptions*  (A1)–(A2)  *, also the reliability estimate*

(3.5) $${\left\|\phi -\phi_{h}\right\|}_{{\overset{˜}{H}}^{-1/2}\left(\Gamma_{}\right)}\leq C_{\mathrm{rel}}\;\eta_{h}$$

*holds. The constant* $C_{\mathrm{eff}}>0$ *depends only on* V*, while* $C_{\mathrm{rel}}>0$ *depends additionally on* $\Gamma_{}$*,* m*,* $\kappa \left(T_{h}\right)$*, and* q*.*

Remark 3.2 The proof reveals that the efficiency estimate  (3.4) is valid for *any* approximation $\phi_{h}$ of ϕ, while the upper reliability estimate  (3.5) requires some Galerkin orthogonality.

Sketch of the proof of Theorem 3.1 Since V is an isomorphism, the residual $r_{h}≔f-V\phi_{h}=V\left(\phi -\phi_{h}\right)$ satisfies ${\left\|r_{h}\right\|}_{H^{1/2}\left(\Gamma \right)}≃{\left\|\phi -\phi_{h}\right\|}_{{\overset{˜}{H}}^{-1/2}\left(\Gamma \right)}$, where the hidden constant depends only on V. Therefore, it suffices to prove (3.4)–(3.5) with ${\left\|\phi -\phi_{h}\right\|}_{{\overset{˜}{H}}^{-1/2}\left(\Gamma \right)}$ replaced by ${\left\|r_{h}\right\|}_{H^{1/2}\left(\Gamma \right)}$. For $u=r_{h}$, Proposition 3.3 proves $\sum_{z\in N_{h}}{\left|r_{h}\right|}_{H^{1/2}\left(\omega_{h}\left(z\right)\right)}^{2}\leq 2{\left\|r_{h}\right\|}_{H^{1/2}\left(\Gamma \right)}^{2}$, and hence results in (3.4). For $u=r_{h}$, Lemma 3.4 shows that it suffices to verify $\sum_{T\in T_{h}}h_{T}^{-1}{\left\|u\right\|}_{L^{2}\left(T\right)}^{2}≲\sum_{z\in N_{h}}{\left|u\right|}_{H^{1/2}\left(\omega_{h}\left(z\right)\right)}^{2}$ to conclude reliability (3.5). This is done via a generalized Poincaré inequality, which involves the abstract assumptions (A1)–(A2) and requires that the residual $u=r_{h}$ is $L^{2}$-orthogonal to the functions $\psi_{T}$; see Lemma 3.6 which is proved by induction on m. Combining Lemmas 3.4 and 3.6, Proposition 3.7 concludes that ${\left\|r_{h}\right\|}_{H^{1/2}\left(\Gamma \right)}^{2}≲\sum_{z\in N_{h}}{\left|r_{h}\right|}_{H^{1/2}\left(\omega_{h}\left(z\right)\right)}^{2}$ and hence results in reliability (3.5).  □

### 3.2 Proof of efficiency estimate  (3.4)

The elementary proof of the following proposition is already found in  [17, page 208], but we include the proof for closed Γ=∂Ω to stress the explicit constant. For open Γ⫋∂Ω, the proof works analogously.

Proposition 3.3 *For arbitrary* $u\in H^{1/2}\left(\Gamma_{}\right)$*, it holds*

(3.6) $$\underset{z\in N_{h}}{\sum }\overset{2}{\underset{H^{1/2}\left(\omega_{h}\left(z\right)\right)}{\left|u\right|}}\leq 2\;{\left\|u\right\|}_{H^{1/2}\left(\Gamma_{}\right)}^{2}\text{.}$$

Proof for closed Γ=∂Ω For x,y∈Γ we abbreviate $U\left(x,y\right)≔{\left|u\left(x\right)-u\left(y\right)\right|}^{2}/{\left|x-y\right|}^{2}$. For $T_{j}=T\in T_{h}$ with some j∈{1,…,n}, we set $T^{+}≔T_{j+1}$. There holds

$$\underset{z\in N_{h}}{\sum }|u\overset{2}{\underset{\omega_{h}\left(z\right)}{|}}=\underset{T\in T_{h}}{\sum }|u\overset{2}{\underset{H^{1/2}\left(T\cup T^{+}\right)}{|}}=\underset{T\in T_{h}}{\sum }\left(\int_{T}\int_{T}U\left(x,y\right)\;dx\;dy+\int_{T}\int_{T^{+}}U\left(x,y\right)\;dx\;dy\right)+\underset{T\in T_{h}}{\sum }\left(\int_{T^{+}}\int_{T^{+}}U\left(x,y\right)\;dx\;dy+\int_{T}\int_{T^{+}}U\left(x,y\right)\;dx\;dy\right)\leq 2\underset{T\in T_{h}}{\sum }\int_{T}\int_{\Gamma }U\left(x,y\right)\;dx\;dy\text{.}$$

The last term is just $2{\left\|u\right\|}_{H^{1/2}\left(\Gamma \right)}^{2}$, which concludes the proof.  □

Proof of Theorem 3.1, eq. (3.4) Recall that ${\left\|r_{h}\right\|}_{H^{1/2}\left(\Gamma_{}\right)}≃{\left\|\phi -\phi_{h}\right\|}_{{\overset{˜}{H}}^{-1/2}\left(\Gamma_{}\right)}$, where the hidden constants depend only on V. Together with  (3.6), this proves  (3.4).  □

### 3.3 Proof of reliability estimate  (3.5)

We start with the following lemma. For the elementary (but long) proof, we refer to  [17, Lemma 2.3]. A detailed proof is also found in  [22, Proposition 2.13].

Lemma 3.4 *There exists a constant* $C_{1}>0$ *such that for all* $u\in H^{1/2}\left(\Gamma_{}\right)$

$${\left\|u\right\|}_{H^{1/2}\left(\Gamma_{}\right)}^{2}\leq \underset{z\in N_{h}}{\sum }\overset{2}{\underset{H^{1/2}\left(\omega_{h}\left(z\right)\right)}{\left|u\right|}}+C_{1}\underset{T\in T_{h}}{\sum }\overset{-1}{\underset{T}{h}}{\left\|u\right\|}_{L^{2}\left(T\right)}^{2}\text{,}$$

*The constant depends only on* $\Gamma_{}$ *and* $\kappa \left(T_{h}\right)$*.*

Our next goal is to bound $\sum_{T\in T_{h}}h_{T}^{-1}{\left\|u\right\|}_{L^{2}\left(T\right)}^{2}$. To this end, we need the following Poincaré-type inequality from  [17, Lemma 2.5].

Lemma 3.5 *Let* I⊂R *be a finite interval with length* |I|>0 *. Then, there holds*

$${\left\|u\right\|}_{L^{2}\left(I\right)}^{2}\leq \frac{\left|I\right|}{2}{\left|u\right|}_{H^{1/2}\left(I\right)}^{2}+\frac{1}{\left|I\right|}{\left|\int_{I}u\left(t\right)\;dt\right|}^{2}\;\text{for all}\;u\in H^{1/2}\left(I\right)\text{.}$$

Lemma 3.6 *Suppose the assumptions*   (A1)–(A2)  *. Let* $u\in H^{1/2}\left(\Gamma \right)$ *satisfy*

(3.7) $$\int_{\Gamma_{}}u\left(x\right)\psi_{T}\left(x\right)\;dx=0\;\text{for all}\;T\in T_{h}\text{.}$$

*Then, there exists a constant* $C_{2}>0$ *which depends only on* $\Gamma_{}$*,* m*,* $\kappa \left(T_{h}\right)$*, and* q *such that for all* $T\in T_{h}$

$${\left\|u\right\|}_{L^{2}\left(T\right)}^{2}\leq C_{2}h_{T}{\left|u\right|}_{H^{1/2}\left(T\right)}^{2}\;\text{if }m=0\text{,}$$

(3.8) $${\left\|u\right\|}_{L^{2}\left(\mathrm{supp}\left(\psi_{T}\right)\right)}^{2}\leq C_{2}\left|\mathrm{supp}\left(\psi_{T}\right)\right|\underset{z\in \omega_{h}^{m-1}\left(T\right)\cap N_{h}}{\sum }\overset{2}{\underset{H^{1/2}\left(\omega_{h}\left(z\right)\right)}{\left|u\right|}}\;\text{if }m>0\text{.}$$

Proof of Lemma 3.6 for closed $\Gamma_{}=\partial \Omega$ The assertion is formulated on the boundary itself. Without loss of generality, we may therefore assume that $\gamma =\gamma_{L}$. Since $\mathrm{supp}\left(\psi_{T}\right)$ is connected, there is an interval I of length |I|≤L with $\gamma \left(I\right)=\mathrm{supp}\left(\psi_{T}\right)$. We use Lemma 3.5 and get

$${\left\|u\circ \gamma \right\|}_{L^{2}\left(I\right)}^{2}\leq \frac{\left|I\right|}{2}{\left|u\circ \gamma \right|}_{H^{1/2}\left(I\right)}^{2}+\frac{1}{\left|I\right|}{\left|\int_{I}u\circ \gamma \left(t\right)\;dt\right|}^{2}\text{.}$$

With the orthogonality (3.7) and Assumption (A2), we see

$${\left|\int_{I}u\circ \gamma \left(t\right)\;dt\right|}^{2}={\left|\int_{\mathrm{supp}\left(\psi_{T}\right)}u\left(y\right)\left(1-\psi_{T}\left(y\right)\right)\;dy\right|}^{2}={\left|\int_{I}\left(u\circ \gamma \left(t\right)\right)\left(1-\psi_{T}\circ \gamma \left(t\right)\right)\;dt\right|}^{2}\leq {\left\|1-\left(\psi_{T}\circ \gamma \right)\right\|}_{L^{2}\left(I\right)}^{2}{\left\|u\circ \gamma \right\|}_{L^{2}\left(I\right)}^{2}\leq \left(1-q\right)\left|I\right|{\left\|u\circ \gamma \right\|}_{L^{2}\left(I\right)}^{2}\text{.}$$

Using the last two inequalities, we therefore get

$${\left\|u\circ \gamma \right\|}_{L^{2}\left(I\right)}^{2}\leq \frac{\left|I\right|}{2}{\left|u\circ \gamma \right|}_{H^{1/2}\left(I\right)}^{2}+\left(1-q\right){\left\|u\circ \gamma \right\|}_{L^{2}\left(I\right)}^{2}\text{.}$$

Together with $\left|I\right|=\left|\gamma \left(I\right)\right|=\left|\mathrm{supp}\left(\psi_{T}\right)\right|$, this implies

(3.9) $${\left\|u\right\|}_{L^{2}\left(\mathrm{supp}\left(\psi_{T}\right)\right)}^{2}\leq \frac{\left|\mathrm{supp}\left(\psi_{T}\right)\right|}{2q}\;{\left|u\circ \gamma \right|}_{H^{1/2}\left(I\right)}^{2}\text{.}$$

For m=0, (A1) with $\left|\mathrm{supp}\left(\psi_{T}\right)\right|=h_{T}$, (2.5) (applicable because of (2.6)), and (3.9) conclude the proof with $C_{2}=C_{\Gamma }^{2}/2q$. To estimate ${\left|u\circ \gamma \right|}_{H^{1/2}\left(I\right)}^{2}$ for m>0, we use induction on ℓ to prove the following assertion for all ℓ∈N:

(3.10) $$\forall j\in Z\;{\left|u\circ \gamma \right|}_{H^{1/2}\left(\left[{\overset{ˇ}{z}}_{j-1},{\overset{ˇ}{z}}_{j+\ell }\right]\right)}^{2}\leq {\left(1+2\kappa \left(T_{h}\right)\right)}^{\ell -1}\overset{j+\ell -1}{\underset{k=j}{\sum }}{\left|u\circ \gamma \right|}_{H^{1/2}\left({\overset{ˇ}{T}}_{k}\cup {\overset{ˇ}{T}}_{k+1}\right)}^{2}\text{.}$$

For ℓ=1, (3.10) even holds with equality. The induction hypothesis for ℓ−1≥1 is

(3.11) $$\forall j\in Z\;{\left|u\circ \gamma \right|}_{H^{1/2}\left(\left[{\overset{ˇ}{z}}_{j-1},{\overset{ˇ}{z}}_{j+\ell -1}\right]\right)}^{2}\leq {\left(1+2\kappa \left(T_{h}\right)\right)}^{\ell -2}\overset{j+\ell -2}{\underset{k=j}{\sum }}{\left|u\circ \gamma \right|}_{H^{1/2}\left({\overset{ˇ}{T}}_{k}\cup {\overset{ˇ}{T}}_{k+1}\right)}^{2}\text{.}$$

For r,s∈R, let

$$\overset{ˇ}{U}\left(r,s\right)≔\frac{{\left|u\left(\gamma \left(r\right)\right)-u\left(\gamma \left(s\right)\right)\right|}^{2}}{{\left|r-s\right|}^{2}}\text{.}$$

For j∈Z, the definition of the Sobolev–Slobodeckij seminorm (2.2) shows

(3.12) $${\left|u\circ \gamma \right|}_{H^{1/2}\left(\left[{\overset{ˇ}{z}}_{j-1},{\overset{ˇ}{z}}_{j+\ell }\right]\right)}^{2}=\int_{\left[{\overset{ˇ}{z}}_{j-1},{\overset{ˇ}{z}}_{j+\ell -1}\right]}\int_{\left[{\overset{ˇ}{z}}_{j-1},{\overset{ˇ}{z}}_{j+\ell -1}\right]}\overset{ˇ}{U}\left(r,s\right)\;dr\;ds+\int_{\left[{\overset{ˇ}{z}}_{j+\ell -1},{\overset{ˇ}{z}}_{j+\ell }\right]}\int_{\left[{\overset{ˇ}{z}}_{j+\ell -1},{\overset{ˇ}{z}}_{j+\ell }\right]}\overset{ˇ}{U}\left(r,s\right)\;dr\;ds+2\int_{\left[{\overset{ˇ}{z}}_{j+\ell -1},{\overset{ˇ}{z}}_{j+\ell }\right]}\int_{\left[{\overset{ˇ}{z}}_{j-1},{\overset{ˇ}{z}}_{j+\ell -1}\right]}\overset{ˇ}{U}\left(r,s\right)\;dr\;ds={\left|u\circ \gamma \right|}_{H^{1/2}\left(\left[{\overset{ˇ}{z}}_{j-1},{\overset{ˇ}{z}}_{j+\ell -1}\right]\right)}^{2}+{\left|u\circ \gamma \right|}_{H^{1/2}\left(\left[{\overset{ˇ}{z}}_{j+\ell -1},{\overset{ˇ}{z}}_{j+\ell }\right]\right)}^{2}+2\int_{\left[{\overset{ˇ}{z}}_{j+\ell -1},{\overset{ˇ}{z}}_{j+\ell }\right]}\int_{\left[{\overset{ˇ}{z}}_{j+\ell -2},{\overset{ˇ}{z}}_{j+\ell -1}\right]}\overset{ˇ}{U}\left(r,s\right)\;dr\;ds+\int_{\left[{\overset{ˇ}{z}}_{j+\ell -1},{\overset{ˇ}{z}}_{j+\ell }\right]}\int_{\left[{\overset{ˇ}{z}}_{j-1},{\overset{ˇ}{z}}_{j+\ell -2}\right]}\overset{ˇ}{U}\left(r,s\right)\;dr\;ds\leq {\left|u\circ \gamma \right|}_{H^{1/2}\left(\left[{\overset{ˇ}{z}}_{j-1},{\overset{ˇ}{z}}_{j+\ell -1}\right]\right)}^{2}+{\left|u\circ \gamma \right|}_{H^{1/2}\left(\left[{\overset{ˇ}{z}}_{j+\ell -2},{\overset{ˇ}{z}}_{j+\ell }\right]\right)}^{2}+2\int_{\left[{\overset{ˇ}{z}}_{j+\ell -1},{\overset{ˇ}{z}}_{j+\ell }\right]}\int_{\left[{\overset{ˇ}{z}}_{j-1},{\overset{ˇ}{z}}_{j+\ell -2}\right]}\overset{ˇ}{U}\left(r,s\right)\;dr\;ds\text{.}$$

For r<t<s∈R, we have

$$\overset{ˇ}{U}\left(r,s\right)\leq 2\frac{{\left|u\left(\gamma \left(r\right)\right)-u\left(\gamma \left(t\right)\right)\right|}^{2}}{{\left|r-s\right|}^{2}}+2\frac{{\left|u\left(\gamma \left(t\right)\right)-u\left(\gamma \left(s\right)\right)\right|}^{2}}{{\left|r-s\right|}^{2}}\leq 2\overset{ˇ}{U}\left(r,t\right)+2\overset{ˇ}{U}\left(t,s\right)\text{.}$$

With the abbreviate notation $h_{k}≔h_{{\overset{ˇ}{T}}_{k}}$, it hence follows

$$\int_{\left[{\overset{ˇ}{z}}_{j+\ell -1},{\overset{ˇ}{z}}_{j+\ell }\right]}\int_{\left[{\overset{ˇ}{z}}_{j-1},{\overset{ˇ}{z}}_{j+\ell -2}\right]}\overset{ˇ}{U}\left(r,s\right)\;dr\;ds=\frac{1}{h_{j+\ell -1}}\int_{\left[{\overset{ˇ}{z}}_{j+\ell -2},{\overset{ˇ}{z}}_{j+\ell -1}\right]}\int_{\left[{\overset{ˇ}{z}}_{j+\ell -1},{\overset{ˇ}{z}}_{j+\ell }\right]}\int_{\left[{\overset{ˇ}{z}}_{j-1},{\overset{ˇ}{z}}_{j+\ell -2}\right]}\overset{ˇ}{U}\left(r,s\right)\;dr\;ds\;dt\leq \frac{2}{h_{j+\ell -1}}\int_{\left[{\overset{ˇ}{z}}_{j+\ell -2},{\overset{ˇ}{z}}_{j+\ell -1}\right]}\int_{\left[{\overset{ˇ}{z}}_{j-1},{\overset{ˇ}{z}}_{j+\ell -2}\right]}\overset{ˇ}{U}\left(r,t\right)\int_{\left[{\overset{ˇ}{z}}_{j+\ell -1},{\overset{ˇ}{z}}_{j+\ell }\right]}1\;ds\;dr\;dt+\frac{2}{h_{j+\ell -1}}\int_{\left[{\overset{ˇ}{z}}_{j+\ell -2},{\overset{ˇ}{z}}_{j+\ell -1}\right]}\int_{\left[{\overset{ˇ}{z}}_{j+\ell -1},{\overset{ˇ}{z}}_{j+\ell }\right]}\overset{ˇ}{U}\left(t,s\right)\int_{\left[{\overset{ˇ}{z}}_{j-1},{\overset{ˇ}{z}}_{j+\ell -2}\right]}1\;dr\;ds\;dt\leq \frac{h_{j+\ell }}{h_{j+\ell -1}}{\left|u\circ \gamma \right|}_{H^{1/2}\left(\left[{\overset{ˇ}{z}}_{j-1},{\overset{ˇ}{z}}_{j+\ell -1}\right]\right)}^{2}+\frac{{\overset{ˇ}{z}}_{j+\ell -2}-{\overset{ˇ}{z}}_{j-1}}{h_{j+\ell -1}}{\left|u\circ \gamma \right|}_{H^{1/2}\left({\overset{ˇ}{T}}_{j+\ell -1}\cup {\overset{ˇ}{T}}_{j+\ell }\right)}^{2}\text{.}$$

There holds

$$\frac{{\overset{ˇ}{z}}_{j+\ell -2}-{\overset{ˇ}{z}}_{j-1}}{h_{j+\ell -1}}=\overset{j+\ell -2}{\underset{k=j}{\sum }}\frac{h_{k}}{h_{j+\ell -1}}\leq \overset{j+\ell -2}{\underset{k=j}{\sum }}\kappa {\left(T_{h}\right)}^{j+\ell -1-k}=\overset{\ell -1}{\underset{k=1}{\sum }}\kappa {\left(T_{h}\right)}^{k}\text{.}$$

This implies

$$\int_{\left[{\overset{ˇ}{z}}_{j+\ell -1},{\overset{ˇ}{z}}_{j+\ell }\right]}\int_{\left[{\overset{ˇ}{z}}_{j-1},{\overset{ˇ}{z}}_{j+\ell -2}\right]}\overset{ˇ}{U}\left(r,s\right)\;dr\;ds\;\leq \kappa \left(T_{h}\right){\left|u\circ \gamma \right|}_{H^{1/2}\left(\left[{\overset{ˇ}{z}}_{j-1},{\overset{ˇ}{z}}_{j+\ell -1}\right]\right)}^{2}+{\left|u\circ \gamma \right|}_{H^{1/2}\left({\overset{ˇ}{T}}_{j+\ell -1}\cup {\overset{ˇ}{T}}_{j+\ell }\right)}^{2}\overset{\ell -1}{\underset{k=1}{\sum }}\kappa {\left(T_{h}\right)}^{k}\text{.}$$

Inserting this into (3.12) and using

$$1+2\overset{\ell -1}{\underset{k=1}{\sum }}\kappa {\left(T_{h}\right)}^{k}\leq {\left(1+2\kappa \left(T_{h}\right)\right)}^{\ell -1}$$

as well as the induction hypothesis (3.11), we obtain

$${\left|u\circ \gamma \right|}_{H^{1/2}\left(\left[{\overset{ˇ}{z}}_{j-1},{\overset{ˇ}{z}}_{j+\ell }\right]\right)}^{2}\leq \left(1+2\kappa \left(T_{h}\right)\right){\left|u\circ \gamma \right|}_{H^{1/2}\left(\left[{\overset{ˇ}{z}}_{j-1},{\overset{ˇ}{z}}_{j+\ell -1}\right]\right)}^{2}+{\left(1+2\kappa \left(T_{h}\right)\right)}^{\ell -1}{\left|u\circ \gamma \right|}_{H^{1/2}\left({\overset{ˇ}{T}}_{j+\ell -1}\cup {\overset{ˇ}{T}}_{j+\ell }\right)}^{2}\leq {\left(1+2\kappa \left(T_{h}\right)\right)}^{\ell -1}\overset{j+\ell -2}{\underset{k=j}{\sum }}{\left|u\circ \gamma \right|}_{H^{1/2}\left({\overset{ˇ}{T}}_{k}\cup {\overset{ˇ}{T}}_{k+1}\right)}^{2}+{\left(1+2\kappa \left(T_{h}\right)\right)}^{\ell -1}{\left|u\circ \gamma \right|}_{H^{1/2}\left({\overset{ˇ}{T}}_{j+\ell -1}\cup {\overset{ˇ}{T}}_{j+\ell }\right)}^{2}={\left(1+2\kappa \left(T_{h}\right)\right)}^{\ell -1}\overset{j+\ell -1}{\underset{k=j}{\sum }}{\left|u\circ \gamma \right|}_{H^{1/2}\left({\overset{ˇ}{T}}_{k}\cup {\overset{ˇ}{T}}_{k+1}\right)}^{2}\text{.}$$

This concludes the induction step and thus proves (3.10). Since Γ=∂Ω is closed, the patch $\omega_{h}^{m}\left(T\right)$ consists either of 2m+1 elements or coincides with Γ, wherefore there is an index j∈Z with

(3.13) $$\gamma \left(\left[{\overset{ˇ}{z}}_{j-1},{\overset{ˇ}{z}}_{\min\left\{j+2m\;,\;j-1+n\right\}}\right]\right)=\omega_{h}^{m}\left(T\right)\text{.}$$

Because of Assumption (A1), one can choose I such that $I\subseteq \left[{\overset{ˇ}{z}}_{j-1},{\overset{ˇ}{z}}_{\min\left\{j+2m\;,\;j-1+n\right\}}\right]$. We use (3.9) and (3.10) for ℓ=min{2m,n−1} to see

$${\left\|u\right\|}_{L^{2}\left(\mathrm{supp}\left(\psi_{T}\right)\right)}^{2}\leq \frac{\left|\mathrm{supp}\left(\psi_{T}\right)\right|}{2q}{\left(1+2\kappa \left(T_{h}\right)\right)}^{\min\left\{2m\;,\;n-1\right\}-1}\overset{\min\left\{j+2m\;,\;j-1+n\right\}-1}{\underset{k=j}{\sum }}{\left|u\circ \gamma \right|}_{H^{1/2}\left({\overset{ˇ}{T}}_{k}\cup {\overset{ˇ}{T}}_{k+1}\right)}^{2}\leq \frac{\left|\mathrm{supp}\left(\psi_{T}\right)\right|}{2q}{\left(1+2\kappa \left(T_{h}\right)\right)}^{2m-1}\overset{\min\left\{j+2m\;,\;j-1+n\right\}-1}{\underset{k=j}{\sum }}{\left|u\circ \gamma \right|}_{H^{1/2}\left({\overset{ˇ}{T}}_{k}\cup {\overset{ˇ}{T}}_{k+1}\right)}^{2}\text{.}$$

Finally, we use (2.5) and

$$\left\{z_{k}:k=j,\ldots ,\min\left\{j+2m\;,\;j-1+n\right\}-1\right\}\subseteq \overset{m-1}{\underset{h}{\omega }}\left(T\right)\cap N_{h}\text{,}$$

which follows immediately from (3.13), to get

$$\overset{\min\left\{j+2m\;,\;j-1+n\right\}-1}{\underset{k=j}{\sum }}{\left|u\circ \gamma \right|}_{H^{1/2}\left({\overset{ˇ}{T}}_{k}\cup {\overset{ˇ}{T}}_{k+1}\right)}^{2}\leq C_{\Gamma }^{2}\overset{\min\left\{j+2m\;,\;j-1+n\right\}-1}{\underset{k=j}{\sum }}{\left|u\right|}_{H^{1/2}\left(\omega_{h}\left(z_{k}\right)\right)}^{2}\leq C_{\Gamma }^{2}\underset{z\in \omega_{h}^{m-1}\left(T\right)\cap N_{h}}{\sum }\overset{2}{\underset{H^{1/2}\left(\omega_{h}\left(z\right)\right)}{\left|u\right|}}\text{,}$$

which concludes the proof.  □

Proof of Lemma 3.6 for open $\Gamma_{}⫋\partial \Omega$ The proof works essentially as before, where (3.10) now becomes

$$\forall j\in N\left(j+\ell \leq n⟹{\left|u\circ \gamma \right|}_{H^{1/2}\left(\left[{\overset{ˇ}{z}}_{j-1},{\overset{ˇ}{z}}_{j+\ell }\right]\right)}^{2}\leq {\left(1+2\kappa \left(T_{h}\right)\right)}^{\ell -1}\overset{j+\ell -1}{\underset{k=j}{\sum }}{\left|u\circ \gamma \right|}_{H^{1/2}\left({\overset{ˇ}{T}}_{k}\cup {\overset{ˇ}{T}}_{k+1}\right)}^{2}\right)\text{.}$$

Details are found in  [22, Lemma 2.15].  □

Proposition 3.7 *Suppose the assumptions*  (A1)*–*(A2)  *and let* $u\in H^{1/2}\left(\Gamma_{}\right)$ *satisfy*   (3.7)*. Then, there exists a constant* $C_{3}>0$ *which depends only on* $\Gamma_{}$*,* m*,* $\kappa \left(T_{h}\right)$*, and* q *such that*

(3.14) $${\left\|u\right\|}_{H^{1/2}\left(\Gamma_{}\right)}^{2}\leq C_{3}\;\underset{z\in N_{h}}{\sum }\overset{2}{\underset{H^{1/2}\left(\omega_{h}\left(z\right)\right)}{\left|u\right|}}\text{.}$$

Proof of Proposition 3.7 for closed $\Gamma_{}=\partial \Omega$ Without loss of generality, we may assume that $\gamma =\gamma_{L}$. Due to Lemma 3.4, it remains to estimate the term $\sum_{T\in T_{h}}h_{T}^{-1}{\left\|u\right\|}_{L^{2}\left(T\right)}^{2}$. For m=0, we see

$$C_{2}^{-1}\underset{T\in T_{h}}{\sum }\overset{-1}{\underset{T}{h}}{\left\|u\right\|}_{L^{2}\left(T\right)}^{2}\leq \underset{T\in T_{h}}{\sum }\overset{2}{\underset{H^{1/2}\left(T\right)}{\left|u\right|}}\leq \underset{z\in N_{h}}{\sum }\overset{2}{\underset{H^{1/2}\left(\omega_{h}\left(z\right)\right)}{\left|u\right|}}\text{.}$$

For m>0, Assumption (A1) and Lemma 3.6 give

(3.15) $${\left\|u\right\|}_{L^{2}\left(T\right)}^{2}\leq {\left\|u\right\|}_{L^{2}\left(\mathrm{supp}\left(\psi_{T}\right)\right)}^{2}\leq C_{2}\left|\omega_{h}^{m}\left(T\right)\right|\underset{z\in \omega_{h}^{m-1}\left(T\right)\cap N_{h}}{\sum }\overset{2}{\underset{H^{1/2}\left(\omega_{h}\left(z\right)\right)}{\left|u\right|}}\text{.}$$

Let j∈{1,…,n} with $T=T_{j}$. We extend the mesh data periodically. With the abbreviate notation $h_{\ell }≔h_{{\overset{ˇ}{T}}_{\ell }}$, we see

(3.16) $$\frac{\left|\omega_{h}^{m}\left(T\right)\right|}{h_{T}}\leq \frac{{\overset{ˇ}{z}}_{j+m}-{\overset{ˇ}{z}}_{j-1-m}}{h_{j}}=\overset{m+1}{\underset{\ell =-m+1}{\sum }}\frac{h_{j-1+\ell }}{h_{j}}\leq \overset{m+1}{\underset{\ell =-m+1}{\sum }}\kappa {\left(T_{h}\right)}^{\left|\ell -1\right|}\text{.}$$

Combining (3.15) and (3.16), we obtain with $C_{3}≔C_{2}\sum_{\ell =-m+1}^{m+1}\kappa {\left(T_{h}\right)}^{\left|\ell -1\right|}$

(3.17) ∑T∈ThhT−1‖u‖L2(T)2≤C3∑T∈Th∑z∈ωhm−1(T)∩Nh|u|H1/2(ωh(z))2=C3∑T∈Th∑z∈Nhz∈ωhm−1(T)|u|H1/2(ωh(z))2=C3∑z∈Nh∑T∈Thz∈ωhm−1(T)|u|H1/2(ωh(z))2=2C3m∑z∈Nh|u|H1/2(ωh(z))2.

This concludes the proof.  □

Proof of Proposition 3.7 for open $\Gamma_{}⫋\partial \Omega$ The proof works essentially as for Γ=∂Ω. For details we refer to  [22, Proposition 2.16].  □

Proof of Theorem 3.1, eq. (3.5) Galerkin BEM ensures the Galerkin orthogonality

$$\int_{\Gamma_{}}r_{h}\left(x\right)u_{h}\left(x\right)\;dx=\int_{\Gamma_{}}\left(V\left(\phi -\phi_{h}\right)\right)\left(x\right)u_{h}\left(x\right)\;dx=0\;\text{for all}\;u_{h}\in X_{h}$$

and hence guarantees  (3.7) for the residual $r_{h}=f-V\phi_{h}=V\left(\phi -\phi_{h}\right)$. Since V is an isomorphism, ${\left\|r_{h}\right\|}_{H^{1/2}\left(\Gamma \right)}≃{\left\|\phi -\phi_{h}\right\|}_{{\overset{˜}{H}}^{-1/2}\left(\Gamma \right)}$ together with  (3.14) proves  (3.5).  □

## 4 Adaptive IGABEM

### 4.1 B-splines and NURBS

Throughout this subsection, we consider *knots* $\overset{ˇ}{K}≔{\left(t_{i}\right)}_{i\in Z}$ on R with $t_{i-1}\leq t_{i}$ for i∈Z and $\lim_{i\to \pm \infty }t_{i}=\pm \infty$. For the multiplicity of any knot $t_{i}$, we write $\#t_{i}$. We denote the corresponding set of *nodes* $\overset{ˇ}{N}≔\left\{t_{i}\;:\;i\in Z\right\}=\left\{{\overset{ˇ}{z}}_{j}\;:\;j\in Z\right\}$ with ${\overset{ˇ}{z}}_{j-1}<{\overset{ˇ}{z}}_{j}$ for j∈Z. For i∈Z, the ith *B-Spline* of degree p is defined inductively by

(4.1) $$\begin{array}{cc} B_{i,0} & ≔\chi_{\left[t_{i-1},t_{i}\right)}\text{,}\\ B_{i,p} & ≔\beta_{i-1,p}B_{i,p-1}+\left(1-\beta_{i,p}\right)B_{i+1,p-1}\;\text{for}\;p\in N\text{,} \end{array}$$

where, for t∈R,

$$\beta_{i,p}\left(t\right)≔\left\{ \begin{array}{cc} \frac{t-t_{i}}{t_{i+p}-t_{i}}\; & \text{if}\;t_{i}\neq t_{i+p}\text{,}\\ 0\; & \text{if}\;t_{i}=t_{i+p}\text{.} \end{array}\right.$$

We also use the notations $B_{i,p}^{\overset{ˇ}{K}}≔B_{i,p}$ and $\beta_{i,p}^{\overset{ˇ}{K}}≔\beta_{i,p}$ to stress the dependence on the knots  $\overset{ˇ}{K}$. The proof of the following theorem is found in  [23, Theorem 6].

Theorem 4.1 *Let* I=[a,b) *be a finite interval and* $p\in N_{0}$ *. Then*

(4.2) $$\left\{B_{i,p}{{|}_{I}\;:\;i\in Z,B_{i,p}|}_{I}\neq 0\right\}$$

*is a basis for the space of all right-continuous* $\overset{ˇ}{N}-$ *piecewise polynomials of degree lower or equal* p *on* I *and which are, at each knot* $t_{i}$*,* $p-\#t_{i}$ *times continuously differentiable if* $p-\#t_{i}\geq 0$*.*

In addition to the knots $\overset{ˇ}{K}={\left(t_{i}\right)}_{i\in Z}$, we consider positive weights $W≔{\left(w_{i}\right)}_{i\in Z}$ with $w_{i}>0$. For i∈Z and $p\in N_{0}$, we define the ith *non-uniform rational B-Spline* of degree p or shortly *NURBS* as

(4.3) $$R_{i,p}≔\frac{w_{i}B_{i,p}}{\underset{\ell \in Z}{\sum }w_{\ell }B_{\ell ,p}}\text{.}$$

We also use the notation $R_{i,p}^{\overset{ˇ}{K},W}≔R_{i,p}$. Note that the denominator is locally finite and never zero as shown in the following lemma.

Lemma 4.2 *For* $p\in N_{0}$ *and* i,ℓ∈Z*, the following assertions hold:*

(i) $R_{i,p}{|}_{\left[t_{\ell -1},t_{\ell }\right)}$ *is a rational function with nonzero denominator, which can be extended continuously at* $t_{\ell }$*.*
(ii) $R_{i,p}$ *vanishes outside the interval* $\left[t_{i-1},t_{i+p}\right)$ *. It is positive on the open interval* $\left(t_{i-1},t_{i+p}\right)$*.*
(iii) *It holds* $t_{i-1}=t_{i+p}$ *if and only if* $R_{i,p}=0$*.*
(iv) $B_{i,p}$ *is completely determined by the* p+2 *knots* $t_{i-1},\ldots ,t_{i+p}$*.* $R_{i,p}$ *is completely determined by the* 3p+2 *knots* $t_{i-p-1},\ldots ,t_{i+2p}$ *and the* 2p+1 *weights* $w_{i-p},\ldots ,w_{i+p}$ *. Therefore, we will also use the notation*
  (4.4) $$R\left(\cdot |t_{i-p-1},\ldots ,t_{i+2p},w_{i-p},\ldots ,w_{i+p}\right)≔R_{i,p}\text{.}$$
(v) *The NURBS functions of degree* p *form a partition of unity, i.e.*
  (4.5) $$\underset{i\in Z}{\sum }R_{i,p}=1\;\text{on}\;R\text{.}$$
(vi) *If all weights are equal, then* $R_{i,p}=B_{i,p}$ *. Hence, B-splines are just special NURBS functions.*
(vii) *Each NURBS function* $R_{i,p}$ *is at least* $p-\#t_{\ell }$ *times continuously differentiable at* $t_{\ell }$ *if* $p-\#t_{\ell }\geq 0$*.*
(viii) *For* s,t∈R *and* c>0*, we have*
  (4.6) $$\forall t\in R\text{:}\;R_{i,p}^{s+\overset{ˇ}{K},W}\left(t\right)=R_{i,p}^{\overset{ˇ}{K},W}\left(t-s\right)$$
  *as well as*
  (4.7) $$\forall t\in R\text{:}\;R_{i,p}^{c\overset{ˇ}{K},W}\left(t\right)=R_{i,p}^{\overset{ˇ}{K},W}\left(t/c\right)\text{.}$$
(ix) *Let* ${\overset{ˇ}{K}}_{\ell }={\left(t_{i,\ell }\right)}_{i\in Z}$ *be a sequence of knots such that* $\#t_{i,\ell }=\#t_{i}$ *for all* i∈Z *and,* $W_{\ell }={\left(w_{i,\ell }\right)}_{i\in Z}$ *a sequence of positive weights. If* ${\left({\overset{ˇ}{K}}_{\ell }\right)}_{\ell \in N}$ *converges pointwise to* $\overset{ˇ}{K}$ *and* ${\left(W_{\ell }\right)}_{\ell \in N}$ *converges pointwise to* W*, then* ${\left(R_{i,p}^{{\overset{ˇ}{K}}_{\ell },W_{\ell }}\right)}_{\ell \in N}$ *converges almost everywhere to* $R_{i,p}^{\overset{ˇ}{K},W}$ *for all* i∈N*.*

Proof The proof for (i)–(v) can be found in  [23, Section 2, page 9–10] for B-splines. The generalization to NURBS is trivial. (vi) is an immediate consequence of (v). (vii) follows from Theorem 4.1. To prove (viii), we note that for all ℓ∈Z and t∈R it holds

$$\chi_{\left[s+t_{\ell -1},s+t_{\ell }\right)}\left(t\right)=\chi_{\left[t_{\ell -1},t_{\ell }\right)}\left(t-s\right)\;\text{and}\;\chi_{\left[ct_{\ell -1},ct_{\ell }+s\right)}\left(t\right)=\chi_{\left[t_{\ell -1},t_{\ell }\right)}\left(t/c\right)$$

as well as

$$\frac{t-\left(s+t_{\ell }\right)}{\left(s+t_{\ell +p}\right)-\left(s+t_{\ell }\right)}=\frac{\left(t-s\right)-t_{\ell }}{t_{\ell +p}-t_{\ell }}\;\text{and}\;\frac{t-ct_{\ell }}{ct_{\ell +p}-ct_{\ell }}=\frac{t/c-t_{\ell }}{t_{\ell +p}-t_{\ell }}\text{.}$$

Hence, the assertion is an immediate consequence of the definition of B-splines. For B-splines, (ix) is proved by induction, noting that for all $p^{\prime }\in N$ and i∈Z, we have

$$\beta_{i,p^{\prime }}^{{\overset{ˇ}{K}}_{\ell }}\overset{a.e.}{⟶}\beta_{i,p^{\prime }}^{\overset{ˇ}{K}}\;\text{and}\;B_{i,0}^{{\overset{ˇ}{K}}_{\ell }}\overset{a.e.}{⟶}B_{i,0}^{\overset{ˇ}{K}}\text{.}$$

This easily implies the convergence of $R_{i,p}^{{\overset{ˇ}{K}}_{\ell }}$.  □

For any $p\in N_{0}$, we define the vector spaces

(4.8) $$S^{p}\left(\overset{ˇ}{K}\right)≔\left\{\underset{i\in Z}{\sum }a_{i}B_{i,p}:a_{i}\in R\right\}$$

as well as

(4.9) $$N^{p}\left(\overset{ˇ}{K},W\right)≔\left\{\underset{i\in Z}{\sum }a_{i}R_{i,p}:a_{i}\in R\right\}=\frac{S^{p}\left(\overset{ˇ}{K}\right)}{\underset{i\in Z}{\sum }w_{i}\overset{\overset{ˇ}{K}}{\underset{i,p}{B}}}\text{.}$$

Note that the sums are locally finite.

An analogous version of the following result is already found in  [17] for the special case of B-splines of degrees p=0,1,2 and knot multiplicity $\#t_{i}=1$ for all i∈Z and weight function φ=1. The following generalization to arbitrary NURBS, however, requires a completely new idea.

Lemma 4.3 *Let* I *be a compact interval with nonempty interior,* $\kappa_{\max}\geq 1$*,* $0<w_{\min}\leq w_{\max}$ *real numbers,* $p\in N_{0}$*, and* $\varphi :I\to R^{+}$ *a piecewise continuously differentiable function with positive infimum. Then there exists a constant*

$$q=q\left(\kappa_{\max},w_{\min},w_{\max},p,\varphi \right)\in \left(0,1\right]$$

*such that for arbitrary knots* $t_{0}\leq \cdots \leq t_{3p+1}\in I$ *and corresponding nodes* ${\overset{ˇ}{z}}_{0},\ldots ,{\overset{ˇ}{z}}_{m}$ *with*

(4.10) $$\kappa \left(t_{0},\ldots ,t_{3p+1}\right)≔\max\left\{\max\left\{\frac{{\overset{ˇ}{z}}_{j+1}-{\overset{ˇ}{z}}_{j}}{{\overset{ˇ}{z}}_{j}-{\overset{ˇ}{z}}_{j-1}},\frac{{\overset{ˇ}{z}}_{j}-{\overset{ˇ}{z}}_{j-1}}{{\overset{ˇ}{z}}_{j+1}-{\overset{ˇ}{z}}_{j}}\right\}:j=1,\ldots ,m-1\right\}\leq \kappa_{\max}\text{,}$$

*weights* $w_{\min}\leq w_{1},\ldots ,w_{2p+1}\leq w_{\max}$ *and all* ℓ∈{p+1,…,2p+1}*,*

(4.11) $${\left\|\left(1-R\left(\cdot |t_{0},\ldots ,t_{3p+1},w_{1},\ldots ,w_{2p+1}\right)\right)\cdot \varphi \right\|}_{L^{1}\left(\left[t_{\ell -1},t_{\ell }\right]\right)}\leq \left(1-q\right){\left\|\varphi \right\|}_{L^{1}\left(\left[t_{\ell -1},t_{\ell }\right]\right)}\text{.}$$

*Note that there holds*

$$\mathrm{supp}\left(R\left(\cdot |t_{0},\ldots ,t_{3p+1},w_{1},\ldots ,w_{2p+1}\right)\right)=\left[t_{p},t_{2p+1}\right]\text{.}$$

Proof We prove the lemma in five steps.

**Step 1:** We give an abstract formulation of the problem. For 1≤ν≤3p+1, we define the bounded set

$$M_{\nu }≔\left\{\left({\overset{ˇ}{z}}_{0},\ldots ,{\overset{ˇ}{z}}_{\nu },w_{1},\ldots ,w_{2p+1}\right)\in I^{\nu }\times {\left[w_{\min},w_{\max}\right]}^{2p+1}:{\overset{ˇ}{z}}_{0}<{\overset{ˇ}{z}}_{1},\forall m\in \left\{2,\ldots ,\nu \right\}:\frac{1}{\kappa_{\max}}\left({\overset{ˇ}{z}}_{m-1}-{\overset{ˇ}{z}}_{m-2}\right)\leq {\overset{ˇ}{z}}_{m}-{\overset{ˇ}{z}}_{m-1}\leq \kappa_{\max}\left({\overset{ˇ}{z}}_{m-1}-{\overset{ˇ}{z}}_{m-2}\right)\right\}\text{.}$$

Note that $\left(\overset{ˇ}{z},w\right)\in M_{\nu }$ already implies ${\overset{ˇ}{z}}_{0}<\cdots <{\overset{ˇ}{z}}_{\nu }$. For a vector of multiplicities $k\in N^{\nu +1}$ with $\sum_{m=0}^{\nu }k_{m}=3p+2$ we introduce the function

$$g_{k,\nu }:R^{\nu }\to R^{3p+2}:\left({\overset{ˇ}{z}}_{0},\ldots ,{\overset{ˇ}{z}}_{\nu }\right)\mapsto \left(\underset{k_{0}\text{-times}}{\underset{︸}{{\overset{ˇ}{z}}_{0},\ldots ,{\overset{ˇ}{z}}_{0}}},\ldots ,\underset{k_{\nu }\text{-times}}{\underset{︸}{{\overset{ˇ}{z}}_{\nu },\ldots ,{\overset{ˇ}{z}}_{\nu }}}\right)\text{.}$$

Moreover, we define for ℓ∈{p+1,…,2p+1} the function

$$\Phi_{k,\ell ,\nu }:M_{\nu }\to R:\left(\overset{ˇ}{z},w\right)\mapsto \frac{{\left\|\left(1-R\left(\cdot |g_{k,\nu }\left(\overset{ˇ}{z}\right),w\right)\right)\cdot \varphi \right\|}_{L^{1}\left(\left[g_{k,\nu }{\left(\overset{ˇ}{z}\right)}_{\ell -1},g_{k,\nu }{\left(\overset{ˇ}{z}\right)}_{\ell }\right]\right)}}{{\left\|\varphi \right\|}_{L^{1}\left(\left[g_{k,\nu }{\left(\overset{ˇ}{z}\right)}_{\ell -1},g_{k,\nu }{\left(\overset{ˇ}{z}\right)}_{\ell }\right]\right)}}\text{,}$$

where $\frac{0}{0}≔0$. Our aim is to show that for arbitrary k,ℓ,ν there holds $\sup\left(\Phi_{k,\ell ,\nu }\left(M_{\nu }\right)\right)<1$. Then, we define the constant (1−q) as the maximum of all these suprema. Note that the maximum is taken over a finite set, since $\sum_{m=0}^{\nu }k_{m}=3p+2$, ℓ∈{p+1,…,2p+1} and 1≤ν≤3p+1. Before we proceed, we show that (1−q) really has the desired properties. Without loss of generality, we can assume that not all considered knots $t_{0},\ldots ,t_{3p+1}$ are equal. The corresponding nodes ${\overset{ˇ}{z}}_{0},\ldots ,{\overset{ˇ}{z}}_{\nu }$ and weights $w_{1},\ldots ,w_{2p+1}$ are in $M_{\nu }$. If k is the corresponding multiplicity vector, (4.11) can indeed be equivalently written as

$$\Phi_{k,\ell ,\nu }\left(\overset{ˇ}{z},w\right)\leq \left(1-q\right)\text{.}$$

**Step 2:** We fix k,ℓ,ν. Without loss of generality, we assume that there exists $0\leq \overset{˜}{\nu }\leq \nu$ such that $\ell -1=\sum_{m=0}^{\overset{˜}{\nu }}k_{m}$. This just means that the appearing integrals have nonempty integration domains $\left[g_{k,\nu }{\left(\overset{ˇ}{z}\right)}_{\ell -1},g_{k,\nu }{\left(\overset{ˇ}{z}\right)}_{\ell }\right]$, since in this case $\Phi_{k,\ell ,\nu }\left(\overset{ˇ}{z},w\right)=0$ is already bounded. Using Lemma 4.2 (ii) and (v), we see that for $\left(\overset{ˇ}{z},w\right)\in M_{\nu }$, the function $R\left(\cdot |g_{k,\nu }\left(\overset{ˇ}{z}\right),w\right)$ attains only values in [0,1] and is positive on the interval $\left(g_{k,\nu }{\left(\overset{ˇ}{z}\right)}_{\ell -1},g_{k,\nu }{\left(\overset{ˇ}{z}\right)}_{\ell }\right)$. This implies

(4.12) $$\Phi_{k,\ell ,\nu }\left(M_{\nu }\right)\subseteq \left[0,1\right)\text{.}$$

Because of Lemma 4.2 (ix), we can apply Lebesgue’s dominated convergence theorem to see that $\Phi_{k,\ell ,\nu }$ is continuous. If $M_{\nu }$ was compact, we would be done. Unfortunately it is not.

**Step 3:** Now, we prove the lemma for φ=1. In the definition of $M_{\nu }$ we replace the interval I by R to define a superset of $M_{\nu }$

$$M_{\nu ,R}≔\left\{\left(\overset{ˇ}{z},w\right)\in R^{\nu }\times {\left[w_{\min},w_{\max}\right]}^{2p+1}:{\overset{ˇ}{z}}_{0}<{\overset{ˇ}{z}}_{1},\forall m\in \left\{2,\ldots ,\nu \right\}:\frac{1}{\kappa_{\max}}\left({\overset{ˇ}{z}}_{m-1}-{\overset{ˇ}{z}}_{m-2}\right)\leq {\overset{ˇ}{z}}_{m}-{\overset{ˇ}{z}}_{m-1}\leq \kappa_{\max}\left({\overset{ˇ}{z}}_{m-1}-{\overset{ˇ}{z}}_{m-2}\right)\right\}\text{.}$$

We extend the function $\Phi_{k,\ell ,\nu }$ to

$${\overset{˜}{\Phi }}_{k,\ell ,\nu }:M_{\nu ,R}\to R:\left(\overset{ˇ}{z},w\right)\mapsto \frac{{\left\|1-R\left(\cdot |g_{k,\nu }\left(\overset{ˇ}{z}\right),w\right)\right\|}_{L^{1}\left(\left[g_{k,\nu }{\left(\overset{ˇ}{z}\right)}_{\ell -1},g_{k,\nu }{\left(\overset{ˇ}{z}\right)}_{\ell }\right]\right)}}{g_{k,\nu }{\left(\overset{ˇ}{z}\right)}_{\ell }-g_{k,\nu }{\left(\overset{ˇ}{z}\right)}_{\ell -1}}\text{.}$$

We define a closed and bounded and hence compact subset of $M_{\nu }$

$$M_{\nu ,R}^{0,1}≔\left\{\left(\overset{ˇ}{z},w\right)\in M_{\nu ,R}\;:\;{\overset{ˇ}{z}}_{0}=0,{\overset{ˇ}{z}}_{1}=1\right\}\text{.}$$

If $\left(\overset{ˇ}{z},w\right)\in M_{\nu ,R}$, then $\left(\frac{\overset{ˇ}{z}-{\overset{ˇ}{z}}_{0}}{{\overset{ˇ}{z}}_{1}-{\overset{ˇ}{z}}_{0}},w\right)\in M_{\nu ,R}^{0,1}$ and due to the substitution rule and Lemma 4.2 (viii), there holds with the notation ${⨏}_{c}^{d}\left(\cdot \right)\left(t\right)\;dt=\int_{c}^{d}\left(\cdot \right)\left(t\right)\;dt/\left(d-c\right)$

$${\overset{˜}{\Phi }}_{k,\ell ,\nu }\left(\overset{ˇ}{z},w\right)={⨏}_{g_{k,\nu }{\left(\overset{ˇ}{z}\right)}_{\ell -1}}^{g_{k,\nu }{\left(\overset{ˇ}{z}\right)}_{\ell }}\left(1-R\left(t\left|g_{k,\nu }\left(\overset{ˇ}{z}\right),w\right)\right)\;dt={⨏}_{\frac{g_{k,\nu }{\left(\overset{ˇ}{z}\right)}_{\ell -1}-{\overset{ˇ}{z}}_{0}}{{\overset{ˇ}{z}}_{1}-{\overset{ˇ}{z}}_{0}}}^{\frac{g_{k,\nu }{\left(\overset{ˇ}{z}\right)}_{\ell }-{\overset{ˇ}{z}}_{0}}{{\overset{ˇ}{z}}_{1}-{\overset{ˇ}{z}}_{0}}}\left(1-R\left(t\left({\overset{ˇ}{z}}_{1}-{\overset{ˇ}{z}}_{0}\right)+{\overset{ˇ}{z}}_{0}\right|g_{k,\nu }\left(\overset{ˇ}{z}\right),w\right)\right)\;dt={\overset{˜}{\Phi }}_{k,\ell ,\nu }\left(\frac{\overset{ˇ}{z}-{\overset{ˇ}{z}}_{0}}{{\overset{ˇ}{z}}_{1}-{\overset{ˇ}{z}}_{0}},w\right)\text{.}$$

Hence we have

$${\overset{˜}{\Phi }}_{k,\ell ,\nu }\left(M_{\nu ,R}\right)={\overset{˜}{\Phi }}_{k,\ell ,\nu }\left(M_{\nu ,R}^{0,1}\right)\text{.}$$

As in Step 2 one sees that ${\overset{˜}{\Phi }}_{k,\ell ,\nu }$ only attains values in [0,1) and is continuous. Since $M_{\nu ,R}^{0,1}$ is compact we get

$$\sup\left(\Phi_{k,\ell ,\nu }\left(M_{\nu }\right)\right)\leq \sup\left({\overset{˜}{\Phi }}_{k,\ell ,\nu }\left(M_{\nu ,R}\right)\right)<1\text{.}$$

This proves the lemma for φ=1.

**Step 4:** We prove the lemma for $\varphi =c_{1}\chi_{\left(-\infty ,T\right)}{{|}_{I}+c_{2}\chi_{\left[T,\infty \right)}|}_{I}$ with $c_{1},c_{2}>0$ and T∈I. Again, we extend the function $\Phi_{k,\ell ,\nu }$ to $M_{\nu ,R}$

$${\overset{˜}{\Phi }}_{k,\ell ,\nu }:M_{\nu ,R}\to R:\left(\overset{ˇ}{z},w\right)\mapsto \frac{{\left\|\left(1-R\left(\cdot |g_{k,\nu }\left(\overset{ˇ}{z}\right),w\right)\right)\left(c_{1}\chi_{\left(-\infty ,T\right)}+c_{2}\chi_{\left[T,\infty \right)}\right)\right\|}_{L^{1}\left(\left[g_{k,\nu }{\left(\overset{ˇ}{z}\right)}_{\ell -1},g_{k,\nu }{\left(\overset{ˇ}{z}\right)}_{\ell }\right]\right)}}{{\left\|c_{1}\chi_{\left(-\infty ,T\right)}+c_{2}\chi_{\left[T,\infty \right)}\right\|}_{L^{1}\left(\left[g_{k,\nu }{\left(\overset{ˇ}{z}\right)}_{\ell -1},g_{k,\nu }{\left(\overset{ˇ}{z}\right)}_{\ell }\right]\right)}}\text{.}$$

For the proof of the lemma, it is sufficient to show $\sup\left({\overset{˜}{\Phi }}_{k,\ell ,\nu }\left(M_{\nu ,R}\right)\right)<1$. Due to the substitution rule and Lemma 4.2 (viii), we can assume without loss of generality that T=0. Because of Step 3 it only remains to show that

$$\sup\left({\overset{˜}{\Phi }}_{k,\ell ,\nu }\left\{\left(\overset{ˇ}{z},w\right)\in M_{\nu ,R}\;:\;{\overset{ˇ}{z}}_{0}\leq 0\leq {\overset{ˇ}{z}}_{\nu }\right\}\right)<1\text{.}$$

As in Step 2, one verifies that ${\overset{˜}{\Phi }}_{k,\ell ,\nu }$ only attains values in [0,1) and is continuous. Moreover, due to the substitution rule and Lemma 4.2 (viii), we have for any element of $\left\{\left(\overset{ˇ}{z},w\right)\in M_{\nu ,R}\;:\;{\overset{ˇ}{z}}_{0}\leq 0\leq {\overset{ˇ}{z}}_{\nu }\right\}$

$${\overset{˜}{\Phi }}_{k,\ell ,\nu }\left(\overset{ˇ}{z},w\right)={\overset{˜}{\Phi }}_{k,\ell ,\nu }\left(\frac{\overset{ˇ}{z}}{{\overset{ˇ}{z}}_{1}-{\overset{ˇ}{z}}_{0}},w\right)$$

and hence

$${\overset{˜}{\Phi }}_{k,\ell ,\nu }\left(\left\{\left(\overset{ˇ}{z},w\right)\in M_{\nu ,R}\;:\;{\overset{ˇ}{z}}_{0}\leq 0\leq {\overset{ˇ}{z}}_{\nu }\right\}\right)={\overset{˜}{\Phi }}_{k,\ell ,\nu }\left(\left\{\left(\overset{ˇ}{z},w\right)\in M_{\nu ,R}\;:\;{\overset{ˇ}{z}}_{1}-{\overset{ˇ}{z}}_{0}=1,{\overset{ˇ}{z}}_{0}\leq 0\leq {\overset{ˇ}{z}}_{\nu }\right\}\right)\text{.}$$

The second set is compact, since it is the image of a closed and bounded set under a continuous mapping. Therefore it attains a maximum smaller than one. This concludes the proof for $\varphi =c_{1}\chi_{\left(-\infty ,T\right)}{{|}_{I}+c_{2}\chi_{\left[T,\infty \right)}|}_{I}$.

**Step 5:** Finally, we are in the position to prove the assertion of the lemma for arbitrary functions φ with the desired properties. Let ${\left(\left({\overset{ˇ}{z}}^{m},w^{m}\right)\right)}_{m\in N}$ be a sequence in $M_{\nu }$ such that the $\Phi_{k,\ell ,\nu }$-values converge to $\sup\left(\Phi_{k,\ell ,\nu }\left(M_{\nu }\right)\right)$. Because of the boundedness of $M_{\nu }$, we can assume convergence of the sequence, where the limit $\left({\overset{ˇ}{z}}^{\infty },w^{\infty }\right)$ is in $\bar{M_{\nu }}$, i.e.  $\left({\overset{ˇ}{z}}^{\infty },w^{\infty }\right)\in M_{\nu }$ or $\left({\overset{ˇ}{z}}^{\infty },w^{\infty }\right)\in I^{\nu }\times {\left[w_{\min},w_{\max}\right]}^{2p+1}$ with ${\overset{ˇ}{z}}_{0}^{\infty }=\cdots ={\overset{ˇ}{z}}_{\nu }^{\infty }$. In the first case, we are done because of (4.12) and the continuity of $\Phi_{k,\ell ,\nu }$. For the second case, we define

$$a_{n}≔g_{k,\nu }{\left({\overset{ˇ}{z}}^{n},w^{n}\right)}_{\ell -1}\text{,}\;b_{n}≔g_{k,\nu }{\left({\overset{ˇ}{z}}^{n},w^{n}\right)}_{\ell }\;\text{and}\;R_{n}≔R\left(\cdot |{\overset{ˇ}{z}}^{n},w^{n}\right)\text{.}$$

Note that $a_{n}<b_{n}$, and that the sequences ${\left(a_{n}\right)}_{n\in N}$ and ${\left(b_{n}\right)}_{n\in N}$ converge to the limit

$$Z≔{\overset{ˇ}{z}}_{0}^{\infty }=\cdots ={\overset{ˇ}{z}}_{\nu }^{\infty }\in I\text{.}$$

We consider two cases.

Case 1: If φ is continuous at the limit Z, it is absolutely continuous on the interval $\left[a_{n},b_{n}\right]$ for sufficiently large n∈N. Hence we have for sufficiently large n∈N

$$\Phi_{k,\ell ,\nu }\left({\overset{ˇ}{z}}^{n},w^{n}\right)=\frac{\int_{a_{n}}^{b_{n}}\left(1-R_{n}\left(t\right)\right)\varphi \left(t\right)\;dt}{\int_{a_{n}}^{b_{n}}\varphi \left(t\right)\;dt}=\frac{\int_{a_{n}}^{b_{n}}\left(1-R_{n}\left(t\right)\right)\left(\varphi \left(a_{n}\right)+\int_{a_{n}}^{t}\varphi^{\prime }\left(\tau \right)\;d\tau \right)\;dt}{\int_{a_{n}}^{b_{n}}\left(\varphi \left(a_{n}\right)+\int_{a_{n}}^{t}\varphi^{\prime }\left(\tau \right)\;d\tau \right)\;dt}\leq \frac{\int_{a_{n}}^{b_{n}}\left(1-R_{n}\left(t\right)\right)\varphi \left(a_{n}\right)\;dt+{\left(b_{n}-a_{n}\right)}^{2}{\left\|\varphi^{\prime }\right\|}_{L^{\infty }\left(I\right)}}{\left(b_{n}-a_{n}\right)\varphi \left(a_{n}\right)-{\left(b_{n}-a_{n}\right)}^{2}{\left\|\varphi^{\prime }\right\|}_{L^{\infty }\left(I\right)}}\text{.}$$

The second summand converges to zero. We consider the first one. For any C∈(0,1), there holds for sufficiently large n∈N

$$\frac{\int_{a_{n}}^{b_{n}}\left(1-R_{n}\left(t\right)\right)\varphi \left(a_{n}\right)\;dt}{\left(b_{n}-a_{n}\right)\varphi \left(a_{n}\right)-{\left(b_{n}-a_{n}\right)}^{2}{\left\|\varphi^{\prime }\right\|}_{L^{\infty }\left(I\right)}}\leq \frac{\int_{a_{n}}^{b_{n}}\left(1-R_{n}\left(t\right)\right)\varphi \left(a_{n}\right)\;dt}{\left(b_{n}-a_{n}\right)\varphi \left(a_{n}\right)\cdot C}\leq \frac{1}{C}\left(1-q\left(\kappa_{\max},w_{\min},w_{\max},p,1\right)\right)\text{.}$$

Since C was arbitrary, this implies

$$\sup\left(\Phi_{k,\ell ,\nu }\left(M_{\nu }\right)\right)\leq \left(1-q\left(\kappa_{\max},w_{\min},w_{\max},p,1\right)\right)<1\text{.}$$

Case 2: If φ is not continuous at the limit Z we proceed as follows. For sufficiently large n∈N, φ is absolutely continuous on $\left[a_{n},Z\right]$ and on $\left[Z,b_{n}\right]$. By considering suitable subsequences, we can assume that $a_{n}<b_{n}\leq Z$, $Z\leq a_{n}<b_{n}$ or $a_{n}\leq Z\leq b_{n}$, each for all n∈N. In the first two cases, we can proceed as in Case 1. In the third case, we argue similarly as in Case 1 to see, with the left-handed limit $\varphi^{\ell }\left(Z\right)$ and the right-handed limit $\varphi^{r}\left(Z\right)$ for n∈N large enough

$$\Phi_{k,\ell ,\nu }\left({\overset{ˇ}{z}}^{n},w^{n}\right)=\frac{\int_{a_{n}}^{b_{n}}\left(1-R_{n}\left(t\right)\right)\varphi \left(t\right)\;dt}{\int_{a_{n}}^{b_{n}}\varphi \left(t\right)\;dt}=\frac{\int_{a_{n}}^{Z}\left(1-R_{n}\left(t\right)\right)\left(\varphi^{\ell }\left(Z\right)-\int_{t}^{Z}\varphi^{\prime }\left(\tau \right)\;d\tau \right)\;dt}{\int_{a_{n}}^{b_{n}}\varphi \left(t\right)\;dt}+\frac{\int_{Z}^{b_{n}}\left(1-R_{n}\left(t\right)\right)\left(\varphi^{r}\left(Z\right)+\int_{Z}^{t}\varphi^{\prime }\left(\tau \right)\;d\tau \right)\;dt}{\int_{a_{n}}^{b_{n}}\varphi \left(t\right)\;dt}\leq \frac{\int_{a_{n}}^{b_{n}}\left(1-R_{n}\left(t\right)\right)\left(\varphi^{\ell }\left(Z\right)\chi_{\left(-\infty ,Z\right)}\left(t\right)+\varphi^{r}\left(Z\right)\chi_{\left[Z,\infty \right)}\left(t\right)\right)\;dt}{\int_{a_{n}}^{b_{n}}\varphi^{\ell }\left(Z\right)\chi_{\left(-\infty ,Z\right)}\left(t\right)+\varphi^{r}\left(Z\right)\chi_{\left[Z,\infty \right)}\left(t\right)\;dt-2{\left(b_{n}-a_{n}\right)}^{2}{\left\|\varphi^{\prime }\right\|}_{L^{\infty }\left(I\right)}}+\frac{2{\left(b_{n}-a_{n}\right)}^{2}{\left\|\varphi^{\prime }\right\|}_{L^{\infty }\left(I\right)}}{\int_{a_{n}}^{b_{n}}\varphi^{\ell }\left(Z\right)\chi_{\left(-\infty ,Z\right)}\left(t\right)+\varphi^{r}\left(Z\right)\chi_{\left[Z,\infty \right)}\left(t\right)\;dt-2{\left(b_{n}-a_{n}\right)}^{2}{\left\|\varphi^{\prime }\right\|}_{L^{\infty }\left(I\right)}}\text{.}$$

Again, the second summand converges to zero, wherefore it remains to consider the first one. For any C∈(0,1), there holds for sufficiently large n∈N

$$\frac{\int_{a_{n}}^{b_{n}}\left(1-R_{n}\left(t\right)\right)\left(\varphi^{\ell }\left(Z\right)\chi_{\left(-\infty ,Z\right)}\left(t\right)+\varphi^{r}\left(Z\right)\chi_{\left[Z,\infty \right)}\left(t\right)\right)\;dt}{\int_{a_{n}}^{b_{n}}\varphi^{\ell }\left(Z\right)\chi_{\left(-\infty ,Z\right)}\left(t\right)+\varphi^{r}\left(Z\right)\chi_{\left[Z,\infty \right)}\left(t\right)\;dt-2{\left(b_{n}-a_{n}\right)}^{2}{\left\|\varphi^{\prime }\right\|}_{L^{\infty }\left(I\right)}}\leq \frac{\int_{a_{n}}^{b_{n}}\left(1-R_{n}\left(t\right)\right)\left(\varphi^{\ell }\left(Z\right)\chi_{\left(-\infty ,Z\right)}\left(t\right)+\varphi^{r}\left(Z\right)\chi_{\left[Z,\infty \right)}\left(t\right)\right)\;dt}{\int_{a_{n}}^{b_{n}}\varphi^{\ell }\left(Z\right)\chi_{\left(-\infty ,Z\right)}\left(t\right)+\varphi^{r}\left(Z\right)\chi_{\left[Z,\infty \right)}\left(t\right)\;dt\cdot C}\leq \frac{1}{C}\left(1-q\left(\kappa_{\max},w_{\min},w_{\max},p,\varphi^{\ell }\left(Z\right)\chi_{\left(-\infty ,Z\right)}{{|}_{I}+\varphi^{r}\left(Z\right)\chi_{\left[Z,\infty \right)}|}_{I}\right)\right)\text{.}$$

Since C was arbitrary, this implies

$$\sup\left(\Phi_{k,\ell ,\nu }\left(M_{\nu }\right)\right)\leq \left(1-q\left(\kappa_{\max},w_{\min},w_{\max},p,\varphi^{\ell }\left(Z\right)\chi_{\left(-\infty ,Z\right)}{{|}_{I}+\varphi^{r}\left(Z\right)\chi_{\left[Z,\infty \right)}|}_{I}\right)\right)<1\text{,}$$

which concludes the proof.  □

We return to our problem (1.1). If Γ=∂Ω is closed, each node $\overset{ˇ}{z}\in {\overset{ˇ}{N}}_{h}$ may be assigned with a multiplicity $\#\overset{ˇ}{z}\leq p+1$. This induces a sequence of non decreasing knots ${\overset{ˇ}{K}}_{h}={\left(t_{i}\right)}_{i=1}^{N}$ on (a,b]. Let $W_{h}={\left(w_{i}\right)}_{i=1}^{N}$ be a sequence of weights on these knots. We extend the knot sequence (b−a)-periodically to ${\left(t_{i}\right)}_{i\in Z}$ and the weight sequence to ${\left(w_{i}\right)}_{i\in Z}$ by $w_{N+i}≔w_{i}$ for i∈Z. For the extended sequences we also write ${\overset{ˇ}{K}}_{h}$ and $W_{h}$. We set

(4.13) $${\overset{ˆ}{N}}^{p}\left({\overset{ˇ}{K}}_{h},W_{h}\right)≔N^{p}\left({\overset{ˇ}{K}}_{h},W_{h}\right){{|}_{\left[a,b\right)}\circ \gamma |}_{\left[a,b\right)}^{-1}\text{.}$$

If $\Gamma_{}\neq \partial \Omega$ is open, we assign to each node $\overset{ˇ}{z}\in {\overset{ˇ}{N}}_{h}$ a corresponding multiplicity $\#\overset{ˇ}{z}\leq p+1$ such that $\#{\overset{ˇ}{z}}_{0}=\#{\overset{ˇ}{z}}_{n}=p+1$. This induces a sequence of non decreasing knots ${\overset{ˇ}{K}}_{h}={\left(t_{i}\right)}_{i=0}^{N}$ on [a,b]. Let $W_{h}={\left(w_{i}\right)}_{i=1}^{N-p}$ be a sequence of weights. To keep the notation simple, we extend the sequences arbitrarily to ${\overset{ˇ}{K}}_{h}={\left(t_{i}\right)}_{i\in Z}$ with $t_{i}\leq t_{i+1}$ for i∈Z, $a>t_{i}\to -\infty$ for i<0 and $b<t_{i}\to \infty$ for i>N, and $W_{h}={\left(w_{i}\right)}_{i\in Z}$ with $w_{i}>0$. This allows to define

(4.14) $${\overset{ˆ}{N}}^{p}\left({\overset{ˇ}{K}}_{h},W_{h}\right)≔N^{p}\left({\overset{ˇ}{K}}_{h},W_{h}\right){|}_{\left[a,b\right]}\circ \gamma^{-1}\text{.}$$

Due to Lemma 4.2 (ii) and (iv), this definition does not depend on how the sequences are extended.

With the following theorem, we conclude that Theorem 3.1 holds for the span of transformed NURBS functions.

Theorem 4.4 *Let* $p\in N_{0}$ *and* m≔⌈p/2⌉ *. Then, the space* $X_{h}≔{\overset{ˆ}{N}}^{p}\left({\overset{ˇ}{K}}_{h},W_{h}\right)$ *is a subspace of* $L^{2}\left(\Gamma_{}\right)$ *which satisfies the assumptions*  (A1)*–*(A2)  *from Section*   3.1   *with the constant of*   Lemma  4.3

$$q=q\left(\kappa \left({\overset{ˇ}{T}}_{h}\right),\min\left(W_{h}\right),\max\left(W_{h}\right),p,\varphi \right)\text{,}$$

*where* $\varphi ={\left|\gamma^{\prime }\right|}_{I}|$ *with* I=[a−(b−a)(m+p),b+(b−a)(2p−m)] *resp.* I=[a,b]*.*

Proof of Theorem 4.4 for closed $\Gamma_{}=\partial \Omega$ Lemma 4.2 (i) and (ii), implies $N^{p}\left({\overset{ˇ}{K}}_{h},W_{h}\right)\subset$$L^{2}\left(R\right)$. This shows ${\overset{ˆ}{N}}^{p}\left({\overset{ˇ}{K}}_{h},W_{h}\right)\subset L^{2}\left(\Gamma_{}\right)$.

Let T be an element of the mesh $T_{h}$, j∈{1,…,n} with $T=T_{j}$, and i∈{1,…,N} with ${\overset{ˇ}{z}}_{j-1}=t_{i-1}$ and ${\overset{ˇ}{z}}_{j}=t_{i}$. We define ${\overset{ˇ}{\psi }}_{T}\left(t\right)≔R_{i-m,p}\left(t\right)$ for t∈[a,b) and extend it continuously at b. We set $\psi_{T}≔{\overset{ˇ}{\psi }}_{T}{{|}_{\left[a,b\right)}\circ \gamma |}_{\left[a,b\right)}^{-1}$. Because of Lemma 4.2 (ii), there holds

(4.15) $${\overset{ˇ}{T}}_{j}\subseteq \left[t_{i-m-1},t_{i-m+p}\right]\cap \left[a,b\right]=\mathrm{supp}\left({\overset{ˇ}{\psi }}_{T}\right)\subseteq \left[{\overset{ˇ}{z}}_{j-m-1},{\overset{ˇ}{z}}_{j-m+p}\right]\subseteq \left[{\overset{ˇ}{z}}_{j-m-1},{\overset{ˇ}{z}}_{j+m}\right]\text{.}$$

Since $\gamma {|}_{\left[a,a+\left(b-a\right)/2\right]}$ and $\gamma {|}_{\left[a+\left(b-a\right)/2,b\right]}$ are homeomorphisms, there holds

(4.16) $$\begin{array}{cc} & \gamma \left(\mathrm{supp}\left({\overset{ˇ}{\psi }}_{T}\right)\right)=\gamma \left(\bar{\left\{t\in \left[a,b\right)\;:\;{\overset{ˇ}{\psi }}_{T}\left(t\right)\neq 0\right\}}\right)=\mathrm{supp}\left(\psi_{T}\right)\text{,} \end{array}$$

wherefore $\mathrm{supp}\left(\psi_{T}\right)$ is connected. With (4.15), this shows

$$T\subseteq \mathrm{supp}\left(\psi_{T}\right)\subseteq \omega_{h}^{m}\left(T\right)\text{,}$$

and hence implies Assumption (A1).

To verify Assumption (A2), we apply Lemma 4.3. Note that $R_{i-m,p}$ is completely determined by the knots in I and their weights. This is due to $I⊇\left[t_{i-m-p-1},t_{i+2p-m}\right]$ and Lemma 4.2 (iv). The regularity constant of these knots from (4.10) is obviously smaller or equal than $\kappa \left({\overset{ˇ}{K}}_{h}\right)$. Since γ is piecewise two times continuously differentiable and its left and right derivative vanishes nowhere, $\left|\gamma^{\prime }\right|$ is piecewise continuously differentiable and is bounded from above by some positive constant. With Lemma 4.3 and (4.16), we hence get

$${\left\|1-\psi_{T}\right\|}_{L^{2}\left(\mathrm{supp}\left(\psi_{T}\right)\right)}^{2}=\int_{\mathrm{supp}\left({\overset{ˇ}{\psi }}_{T}\right)}{\left(1-{\overset{ˇ}{\psi }}_{T}\right)}^{2}\left|\gamma^{\prime }\left(t\right)\right|\;dt\leq \int_{\mathrm{supp}\left({\overset{ˇ}{\psi }}_{T}\right)}\left(1-{\overset{ˇ}{\psi }}_{T}\right)\left|\gamma^{\prime }\left(t\right)\right|\;dt={\left\|\left(1-{\overset{ˇ}{\psi }}_{T}\right)\left|\gamma^{\prime }\right|\right\|}_{L^{1}\left(\left[t_{i-m-1},t_{i-m+p}\right]\cap \left[a,b\right]\right)}\leq \left(1-q\right){\left\|\left|\gamma^{\prime }\right|\right\|}_{L^{1}\left(\left[t_{i-m-1},t_{i-m+p}\right]\cap \left[a,b\right]\right)}=\left(1-q\right)\int_{\mathrm{supp}\left({\overset{ˇ}{\psi }}_{T}\right)}\left|\gamma^{\prime }\left(t\right)\right|\;dt=\left(1-q\right)\left|\mathrm{supp}\left(\psi_{T}\right)\right|\text{.}$$

Consequently, Assumption (A2) is also fulfilled. This concludes the proof.  □

Proof of Theorem 4.4 for open $\Gamma_{}⫋\partial \Omega$ The proof works analogously as before. Details are found in  [22, Theorem 4.14].  □

### 4.2 Knot insertion

Before we formulate an adaptive algorithm based on NURBS, we recall refinement by *knot-insertion*, see e.g.  [23, Section 11]. For general knots $\overset{ˇ}{K}={\left(t_{i}\right)}_{i\in Z}$ as in the previous subsection, a polynomial degree $p\in N_{0}$, and a refined sequence ${\overset{ˇ}{K}}^{\prime }={\left(t_{i}^{\prime }\right)}_{i\in Z}$ (i.e.,  $\overset{ˇ}{K}$ is a subsequence of $\overset{ˇ}{K}$) Theorem 4.1 implies nestedness

(4.17) $$S^{p}\left(\overset{ˇ}{K}\right)\subseteq S^{p}\left({\overset{ˇ}{K}}^{\prime }\right)\text{.}$$

We assume that the multiplicities of the knots in ${\overset{ˇ}{K}}^{\prime }$ are lower or equal p+1. Because of Lemma 4.2 (ii), and Theorem 4.1 each element $\sum_{i\in Z}a_{i}B_{i,p}^{\overset{ˇ}{K}}\in S\left(\overset{ˇ}{K}\right)$ admits some unique coefficient vector ${\left(a_{i}^{\prime }\right)}_{i\in Z}$ with

(4.18) $$\underset{i\in Z}{\sum }a_{i}\overset{\overset{ˇ}{K}}{\underset{i,p}{B}}=\underset{i\in Z}{\sum }\overset{\prime }{\underset{i}{a}}B_{i,p}^{{\overset{ˇ}{K}}^{\prime }}\text{.}$$

If ${\overset{ˇ}{K}}^{\prime }$ contains only one additional knot $t^{\prime }$ (possibly already contained in $\overset{ˇ}{K}$), the coefficients can be calculated explicitly. We assume $t_{i}=t_{i}^{\prime }$ for all i with $t_{i}<t^{\prime }$. Then,  [23, Algorithm 11] shows

(4.19) $$\begin{array}{c} a_{i}^{\prime }=\left\{ \begin{array}{cc} a_{i}\; & \text{if }t_{i+p}\leq t^{\prime }\text{,}\\ \left(1-\beta_{i-1,p}^{\overset{ˇ}{K}}\left(t^{\prime }\right)\right)a_{i-1}+\beta_{i-1,p}^{\overset{ˇ}{K}}\left(t^{\prime }\right)a_{i}\; & \text{if }t_{i}<t^{\prime }<t_{i+p}\text{,}\\ a_{i-1}\; & \text{if }t^{\prime }\leq t_{i}\text{.} \end{array}\right. \end{array}$$

For closed Γ=∂Ω, we consider again knots ${\overset{ˇ}{K}}_{h}={\left(t_{i}\right)}_{i=1}^{N}$ and weights $W_{h}={\left(w_{i}\right)}_{i=1}^{N}$ as in the previous subsection. We additionally assume p+1≤N. Now we insert an additional knot $t^{\prime }\in \left(a,b\right]$ to the knots ${\overset{ˇ}{K}}_{h}$ such that the multiplicities of the new knots ${\overset{ˇ}{K}}_{h}^{\prime }$ are still smaller or equal than p+1. The new knots are extended (b−a)-periodically. We want to find the unique weights ${\left(w_{i}^{\prime }\right)}_{i\in Z}$ which fulfill

(4.20) $$\underset{i\in Z}{\sum }w_{i}\overset{{\overset{ˇ}{K}}_{h}}{\underset{i,p}{B}}=\underset{i\in Z}{\sum }\overset{\prime }{\underset{i}{w}}B_{i,p}^{{\overset{ˇ}{K}}_{h}^{\prime }}\text{.}$$

With (4.9) and (4.17), this already implies nestedness

(4.21) $${\overset{ˆ}{N}}^{p}\left({\overset{ˇ}{K}}_{h},W_{h}\right)\subseteq {\overset{ˆ}{N}}^{p}\left({\overset{ˇ}{K}}_{h}^{\prime },W_{h}^{\prime }\right)\text{.}$$

The sought weights ${\left(w_{i}^{\prime }\right)}_{i\in Z}$ are obviously (N+1)-periodic. We cannot immediately apply (4.19), since infinitely many knots $\left\{t^{\prime }+k\left(b-a\right):k\in Z\right\}$ are added to ${\overset{ˇ}{K}}_{h}$. First, we add $\left\{t^{\prime }+k\left(b-a\right):k\in Z\setminus \left\{-1,0,1\right\}\right\}$ to ${\overset{ˇ}{K}}_{h}$ and obtain ${\overset{ˇ}{K}}^{+}={\left(t_{i}^{+}\right)}_{i\in Z}$ with $t_{0}=t_{0}^{+}$ and $t_{1}=t_{1}^{+}$. There exist unique weights ${\left(w_{i}^{+}\right)}_{i\in Z}$ with

$$\underset{i\in Z}{\sum }w_{i}\overset{{\overset{ˇ}{K}}_{h}}{\underset{i,p}{B}}=\underset{i\in Z}{\sum }\overset{+}{\underset{i}{w}}B_{i,p}^{{\overset{ˇ}{K}}^{+}}\text{.}$$

With $I≔\left[t_{-1},t_{N+1}\right)$, Lemma 4.2 (ii) and (iv), and our assumption p+1≤N imply

$$\overset{N+1}{\underset{i=-p}{\sum }}w_{i}B_{i,p}^{{\overset{ˇ}{K}}_{h}}{|}_{I}=\overset{N+1}{\underset{i=-p}{\sum }}w_{i}^{+}B_{i,p}^{{\overset{ˇ}{K}}^{+}}{|}_{I}=\overset{N+1}{\underset{i=-p}{\sum }}w_{i}^{+}B_{i,p}^{{\overset{ˇ}{K}}_{h}}{|}_{I}\text{.}$$

With $t_{N}<t_{N+1}$, it is easy to check that $B_{i,p}^{{\overset{ˇ}{K}}_{h}}{|}_{I}\neq 0$ for i=0,…,N. Hence, Theorem 4.1 implies $w_{i}=w_{i}^{+}$ for i=0,…,N. It just remains to add the knots $t^{\prime }-\left(b-a\right)$, $t^{\prime }$ and $t^{\prime }+\left(b-a\right)$. To this end, we can repetitively apply (4.19) to obtain the weights ${\left(w_{i}^{\prime }\right)}_{i=1}^{N+1}$. Note that this only involves the weights ${\left(w_{i}^{+}\right)}_{i=0}^{N}$ are needed. Moreover, the new weights ${\left(w_{i}^{\prime }\right)}_{i=1}^{N+1}$ are just convex combinations of the old ones ${\left(w_{i}\right)}_{i=1}^{N}$.

For open Γ⫋∂Ω, a knot $t^{\prime }\in \left(a,b\right]$ can analogously be inserted to the knots ${\overset{ˇ}{K}}_{h}={\left(t_{i}\right)}_{i=0}^{N}$.

### 4.3 Adaptive algorithm

In this section, we introduce an adaptive algorithm, which uses the local contributions of $\eta_{h}$ to steer the h-refinement of the mesh $T_{h}$ as well as the increase of the multiplicity of the nodes $N_{h}$. To respect the iterative character of this procedure, all discrete quantities (as, e.g.,  $T_{h}$, $\phi_{h}$, etc.) are indexed by the level $\ell \in N_{0}$ of the adaptive process instead of the mesh-size h. Let 0<θ<1 be an adaptivity parameter and $p\in N_{0}$ a polynomial degree. We start with some nodes ${\overset{ˇ}{N}}_{0}$. Each node has a multiplicity lower or equal p+1, where for open Γ⫋∂Ω we assume #a=#b=p+1. This induces knots ${\overset{ˇ}{K}}_{0}$. Let $W_{0}$ be some initial positive weights. We assume $p+1\leq N_{0}$ and for closed Γ=∂Ω, |T|≤|Γ|/4 for all $T\in T_{0}$. As the initial trial space, we consider

(4.22) $$X_{0}≔{\overset{ˆ}{N}}^{p}\left({\overset{ˇ}{K}}_{0},W_{0}\right)\subset L^{2}\left(\Gamma_{}\right)\subset H^{-1/2}\left(\Gamma_{}\right)\text{.}$$

The adaptive algorithm with *Dörfler marking* reads as follows:

Algorithm 4.5 **Input:** Adaptivity parameter 0<θ<1, polynomial order $p\in N_{0}$, initial mesh $T_{0}$ with knots ${\overset{ˇ}{K}}_{0}$, initial weights $W_{0}$.

**Adaptive loop:** Iterate the following steps, until $\eta_{\ell }$ is sufficiently small:

(i) Compute discrete solution $\phi_{\ell }\in X_{\ell }$.
(ii) Compute indicators $\eta_{\ell }\left(z\right)$ for all nodes $z\in N_{\ell }$.
(iii) Determine a minimal set of nodes $M_{\ell }\subseteq N_{\ell }$ such that
  (4.23) $$\theta \;\eta_{\ell }^{2}\leq \underset{z\in M_{\ell }}{\sum }\eta_{\ell }{\left(z\right)}^{2}\text{.}$$
(iv) If both nodes of an element $T\in T_{\ell }$ belong to $M_{\ell }$, T will be marked.
(v) For all other nodes in $M_{\ell }$, the multiplicity will be increased if it is smaller than p+1, otherwise the elements which contain one of these nodes $z\in M_{\ell }$, will be marked.
(vi) Refine all marked elements $T\in T_{\ell }$ by bisection of the corresponding $\overset{ˇ}{T}\in {\overset{ˇ}{T}}_{\ell }$. Use further bisections to guarantee that the new mesh $T_{\ell +1}$ satisfies
  (4.24) $$\kappa \left({\overset{ˇ}{T}}_{\ell +1}\right)\leq 2\kappa \left({\overset{ˇ}{T}}_{0}\right)\text{.}$$
  Update counter ℓ↦ℓ+1.

**Output:** Approximate solutions $\phi_{\ell }$ and error estimators $\eta_{\ell }$ for all $\ell \in N_{0}$.

An optimal 1D bisection algorithm which ensures (4.24), is discussed and analyzed in  [24]. Note that boundedness of $\kappa \left({\overset{ˇ}{T}}_{\ell }\right)$ implies as well boundedness of $\kappa \left(T_{\ell }\right)$. Moreover, there holds

(4.25) $$\min\left(W_{0}\right)\leq \min\left(W_{\ell }\right)\leq \max\left(W_{\ell }\right)\leq \max\left(W_{0}\right)\text{,}$$

since the new weights are convex combinations of the old weights. Hence, Theorems 3.1 and 4.4 apply and show efficiency and reliability of the estimator

(4.26) $$C_{\mathrm{rel}}^{-1}\;{\left\|\phi -\phi_{\ell }\right\|}_{{\overset{˜}{H}}^{-1/2}\left(\Gamma_{}\right)}\leq \eta_{\ell }\leq C_{\mathrm{eff}}\;{\left\|\phi -\phi_{\ell }\right\|}_{{\overset{˜}{H}}^{-1/2}\left(\Gamma_{}\right)}\text{.}$$

## 5 Numerical experiments

In this section, we empirically investigate the performance of Algorithm 4.5 in three typical situations: In Sections  5.2 and 5.3, we consider a closed boundary Γ=∂Ω, where the solution is smooth resp. exhibits a generic (i.e., geometry induced) singularity. In Section  5.4, we consider a slit problem. In either example, the exact solution is known and allows us to compute the Galerkin error to underline reliability and efficiency of the proposed estimator.

In each example, the parametrization γ of the part $\Gamma_{}$ of the boundary is a NURBS curve and thus has the special form

(5.1) $$\gamma \left(t\right)=\underset{i\in Z}{\sum }C_{i}\overset{{\overset{ˇ}{K}}_{0},W_{0}}{\underset{i,p}{R}}\left(t\right)$$

for all t∈[a,b]. Here, p∈N is the polynomial degree, ${\overset{ˇ}{K}}_{0}$ and $W_{0}$ are knots and weights as in Section  4.3 and ${\left(C_{i}\right)}_{i\in Z}$ are *control points* in $R^{2}$ which are periodic for closed Γ=∂Ω.

We choose the same polynomial degree p for our approximation spaces $X_{\ell }$. Since for the refinement strategy only knot insertion is used, we can apply (4.17) and (4.20) to see for the first and second component of γ

(5.2) $$\gamma_{1},\gamma_{2}\in N^{p}\left({\overset{ˇ}{K}}_{\ell },W_{\ell }\right){|}_{\left[a,b\right]}\text{.}$$

Hence, this approach reflects the main idea of isogeometric analysis, where the same space is used for the geometry and for the approximation. We compare uniform refinement, where $M_{\ell }=N_{\ell }$ and hence all elements are refined, and adaptive refinement with θ=0.75.

### 5.1 Stable implementation of adaptive IGABEM

To compute the approximation $\phi_{h}$ of one step of the adaptive algorithm, we first note that Theorem 4.1 implies that

(5.3) $$\left\{R_{i,p}{|}_{\left[a,b\right)}:i=\left(1-p\right),\ldots ,N-\#b+1\right\}\circ \gamma {|}_{\left[a,b\right)}^{-1}$$

resp.

(5.4) $$\left\{R_{i,p}{|}_{\left[a,b\right]}:i=1,\ldots ,N\right\}\circ \gamma^{-1}$$

forms a basis of $\overset{ˆ}{N}\left({\overset{ˇ}{K}}_{h},W_{h}\right)$. We abbreviate the elements of this basis with ${\overset{ˆ}{R}}_{i}$ and its index set with I. Then, there holds the unique basis representation $\phi_{h}=\sum_{i\in I}c_{h,i}{\overset{ˆ}{R}}_{i}$. The coefficient vector $c_{h}$ is the unique solution of

(5.5) $$V_{h}c_{h}=f_{h}$$

with the symmetric positive definite matrix

(5.6) $$V_{h}≔{\left({\langle V{\overset{ˆ}{R}}_{j}\;;\;{\overset{ˆ}{R}}_{i}\rangle }_{L^{2}\left(\Gamma \right)}\right)}_{i,j\in I}$$

and the right-hand side vector

(5.7) $$f_{h}≔{\left({\langle f\;;\;{\overset{ˆ}{R}}_{i}\rangle }_{L^{2}\left(\Gamma \right)}\right)}_{i\in I}\text{.}$$

The energy norm then reads

(5.8) $$\left|\;\right|\;\left|\phi_{h}\right|\;{\left|\;\right|}^{2}=\langle V\phi_{h}\;;\;\phi_{h}\rangle =c_{h}^{\;\;T}V_{h}c_{h}\text{.}$$

To calculate $V_{h}$, $f_{h}$ and the $H^{1/2}$-seminorms of the residual $r_{h}=f-V\phi_{h}$, singular integrals and double integrals have to be evaluated. Since this is hardly possible analytically, we approximate the appearing integrals. To this end, we first write them as sum of integrals over the elements of the mesh $\overset{ˇ}{T}$. In the spirit of  [6, Section 5.3], the local integrals which contain singularities, are transformed by *Duffy transformations* such that either the singularity vanishes or a pure logarithmic singularity of the form log(t) on [0,1] remains. Finally, the integrals are evaluated over the domain [0,1] or ${\left[0,1\right]}^{2}$ using tensor-Gauss quadrature with weight function 1 resp. log(t). Since the integrands are smooth up to logarithmic terms, this yields exponential convergence of adapted Gauss quadrature and hence provides accurate approximations. For closed Γ=∂Ω and arbitrary parametrization γ as in Section  2.3, all details are elaborated in  [22, Section 5].

### 5.2 Adaptive IGABEM for problem with smooth solution

Let Ω be the circle with midpoint (0,0) and radius 1/10. We consider the Laplace–Dirichlet problem on Ω

(5.9) $$\begin{array}{c} -\Delta u=0\;\text{in}\;\Omega \;\text{ and }\;u=g\;\text{on}\;\Gamma_{} \end{array}$$

for given Dirichlet data $g\in H^{1/2}\left(\Gamma_{}\right)$ and closed boundary Γ=∂Ω. The problem is equivalent to Symm’s integral equation (1.1) with the *single-layer integral operator*

(5.10) $$V:{\overset{˜}{H}}^{-1/2}\left(\Gamma_{}\right)\to H^{1/2}\left(\Gamma_{}\right)\text{,}\;V\phi \left(x\right)≔-\frac{1}{2\pi }\int_{\Gamma_{}}\log\left(\left|x-y\right|\right)\phi \left(y\right)\;dy$$

and the right-hand side f=(K+1/2)g, where

(5.11) $$K:H^{1/2}\left(\Gamma_{}\right)\to H^{1/2}\left(\Gamma_{}\right)\text{,}\;Kg\left(x\right)≔-\frac{1}{2\pi }\int_{\Gamma_{}}\left(\partial_{\nu \left(y\right)}\log\left(\left|x-y\right|\right)\right)g\left(y\right)\;dy$$

denotes the *double-layer integral operator*. The unique solution of (1.1) is the normal derivative ϕ=∂u/∂ν of the weak solution $u\in H^{1}\left(\Omega \right)$ of (5.9).

We prescribe the exact solution $u\left(x,y\right)=x^{2}+10xy-y^{2}$ and solve Symm’s integral equation  (1.1) on the closed boundary Γ=∂Ω. The normal derivative ϕ=∂u/∂ν reads

$$\phi \left(x,y\right)=20\left(x^{2}+10xy-y^{2}\right)\text{.}$$

The geometry is parametrized on [0,1] by the NURBS curve induced by

p=2,

$${\overset{ˇ}{K}}_{0}=\left(\frac{1}{4},\frac{1}{4},\frac{2}{4},\frac{2}{4},\frac{3}{4},\frac{3}{4},1,1,1\right)\text{,}$$

$$W_{0}=\left(1,\frac{1}{\sqrt{2}},1,\frac{1}{\sqrt{2}},1,\frac{1}{\sqrt{2}},1,1,\frac{1}{\sqrt{2}}\right)\text{,}$$

$${\left(C_{i}\right)}_{i=1}^{N_{0}}=\frac{1}{10}\cdot \left(\left(\begin{array}{c} 0\\ 1 \end{array}\right),\left(\begin{array}{c} -1\\ 1 \end{array}\right),\left(\begin{array}{c} -1\\ 0 \end{array}\right),\left(\begin{array}{c} -1\\ -1 \end{array}\right),\left(\begin{array}{c} 0\\ -1 \end{array}\right),\left(\begin{array}{c} 1\\ -1 \end{array}\right),\left(\begin{array}{c} 1\\ 0 \end{array}\right),\left(\begin{array}{c} 1\\ 0 \end{array}\right),\left(\begin{array}{c} 1\\ 1 \end{array}\right)\right)\text{.}$$

**Fig. 5.1:**
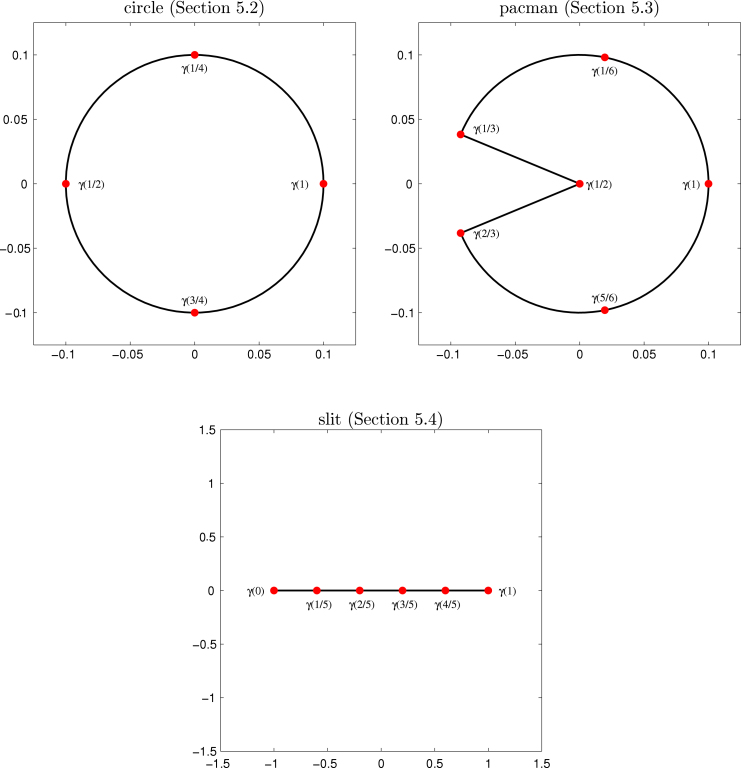
Geometries and initial nodes for the experiments from Sections 5.2–5.4.

**Fig. 5.2:**
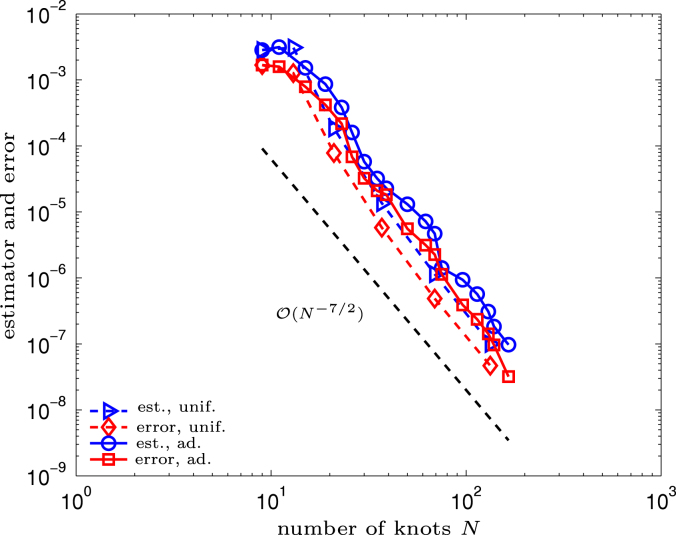
Experiment with smooth solution on circle geometry from Section  5.2. Error and estimator are plotted versus the number of knots  N.

Note that this parametrization does not coincide with the natural parametrization t↦(cos(t),sin(t)). Fig. 5.1 visualizes the geometry and the γ-values of the initial nodes. Fig. 5.2 shows error and error estimator for the uniform and the adaptive approach. All values are plotted in a log–log scale such that the experimental convergence rates are visible as the slope of the corresponding curves. The Galerkin orthogonality allows to compute the energy error by

(5.12) $$\left|\;\right|\;\left|\phi -\phi_{\ell }\right|\;{\left|\;\right|}^{2}=\left|\;\right|\;\left|\phi \right|\;{\left|\;\right|}^{2}-\left|\;\right|\;\left|\phi_{\ell }\right|\;{\left|\;\right|}^{2}=13\pi /5000-\left|\;\right|\;\left|\phi_{\ell }\right|\;{\left|\;\right|}^{2}\text{.}$$

With respect to the number of knots N, both approaches lead to the rate $O\left(N^{-7/2}\right)$. If discontinuous piecewise polynomials of order 2 were used as ansatz space, this is the optimal convergence rate. In each case, the curves for the error and the corresponding estimator are parallel. This empirically confirms the proven efficiency and reliability of the Faermann estimator $\eta_{h}$.

### 5.3 Adaptive IGABEM for problem with generic singularity

As second example, we consider the Laplace–Dirichlet problem (5.9) on the pacman geometry

$$\Omega ≔\left\{r\left(\cos\left(\alpha \right),\sin\left(\alpha \right)\right):0\leq r<\frac{1}{10},\alpha \in \left(-\frac{\pi }{2\tau },\frac{\pi }{2\tau }\right)\right\}\text{,}$$

with τ=4/7; see Fig. 5.1. We prescribe the exact solution

$$u\left(x,y\right)=r^{\tau }\cos\left(\tau \alpha \right)\;\text{in polar coordinates}\;\left(x,y\right)=r\left(\cos\alpha ,\sin\alpha \right)\text{.}$$

The normal derivative of u reads

$$\phi \left(x,y\right)=\left(\begin{array}{c} \cos\left(\alpha \right)\cos\left(\tau \alpha \right)+\sin\left(\alpha \right)\sin\left(\tau \alpha \right)\\ \sin\left(\alpha \right)\cos\left(\tau \alpha \right)-\cos\left(\alpha \right)\sin\left(\tau \alpha \right) \end{array}\right)\cdot \nu \left(x,y\right)\cdot \tau \cdot r^{\tau -1}$$

and has a generic singularity at the origin. With w=cos(π/τ), the geometry is parametrized on [0,1] by the NURBS curve induced by

p=2,

$${\overset{ˇ}{K}}_{0}=\left(\frac{1}{6},\frac{1}{6},\frac{2}{6},\frac{2}{6},\frac{3}{6},\frac{3}{6},\frac{4}{6},\frac{4}{6},\frac{5}{6},\frac{5}{6},1,1,1\right)\text{,}$$

$$W_{0}=\left(1,w,1,1,1,1,1,w,1,w,1,1,w\right)\text{,}$$

$${\left(C_{i}\right)}_{i=1}^{N_{0}}=\frac{1}{10}\cdot \left(\left(\begin{array}{c} \cos\left(\pi /\tau \cdot 2/8\right)\\ \sin\left(\pi /\tau \cdot 2/8\right) \end{array}\right),\frac{1}{w}\left(\begin{array}{c} \cos\left(\pi /\tau \cdot 3/8\right)\\ \sin\left(\pi /\tau \cdot 3/8\right) \end{array}\right),\left(\begin{array}{c} \cos\left(\pi /\tau \cdot 4/8\right)\\ \sin\left(\pi /\tau \cdot 4/8\right) \end{array}\right)\text{,}\;\frac{1}{2}\left(\begin{array}{c} \cos\left(\pi /\tau \cdot 4/8\right)\\ \sin\left(\pi /\tau \cdot 4/8\right) \end{array}\right),\left(\begin{array}{c} 0\\ 0 \end{array}\right),\frac{1}{2}\left(\begin{array}{c} \cos\left(\pi /\tau \cdot \left(-4\right)/8\right)\\ \sin\left(\pi /\tau \cdot \left(-4\right)/8\right) \end{array}\right),\left(\begin{array}{c} \cos\left(\pi /\tau \cdot \left(-4\right)/8\right)\\ \sin\left(\pi /\tau \cdot \left(-4\right)/8\right) \end{array}\right)\text{,}\;\frac{1}{w}\left(\begin{array}{c} \cos\left(\pi /\tau \cdot \left(-3\right)/8\right)\\ \sin\left(\pi /\tau \cdot \left(-3\right)/8\right) \end{array}\right),\left(\begin{array}{c} \cos\left(\pi /\tau \cdot \left(-2\right)/8\right)\\ \sin\left(\pi /\tau \cdot \left(-2\right)/8\right) \end{array}\right),\frac{1}{w}\left(\begin{array}{c} \cos\left(\pi /\tau \cdot \left(-1\right)/8\right)\\ \sin\left(\pi /\tau \cdot \left(-1\right)/8\right) \end{array}\right)\text{,}\;\left(\begin{array}{c} \cos\left(\pi /\tau \cdot 0/8\right)\\ \sin\left(\pi /\tau \cdot 0/8\right) \end{array}\right),\left(\begin{array}{c} \cos\left(\pi /\tau \cdot 0/8\right)\\ \sin\left(\pi /\tau \cdot 0/8\right) \end{array}\right),\frac{1}{w}\left(\begin{array}{c} \cos\left(\pi /\tau \cdot 1/8\right)\\ \sin\left(\pi /\tau \cdot 1/8\right) \end{array}\right)\right)\text{.}$$

**Fig. 5.3:**
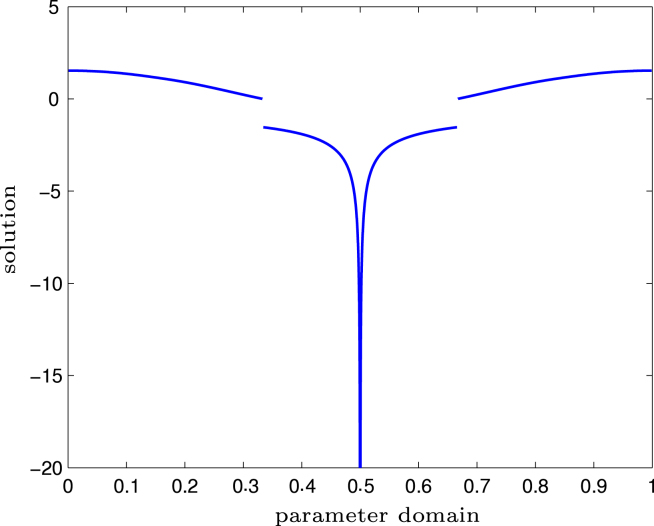
Experiment with singular solution on pacman geometry from Section  5.3. The singular solution ϕ∘γ is plotted on the parameter interval, where 0.5 corresponds to the origin, where ϕ is singular.

In Fig. 5.3, the solution ϕ is plotted over the parameter domain. We can see that ϕ has a singularity at t=1/2 as well as two jumps at t=1/3 resp. t=2/3.

**Fig. 5.4:**
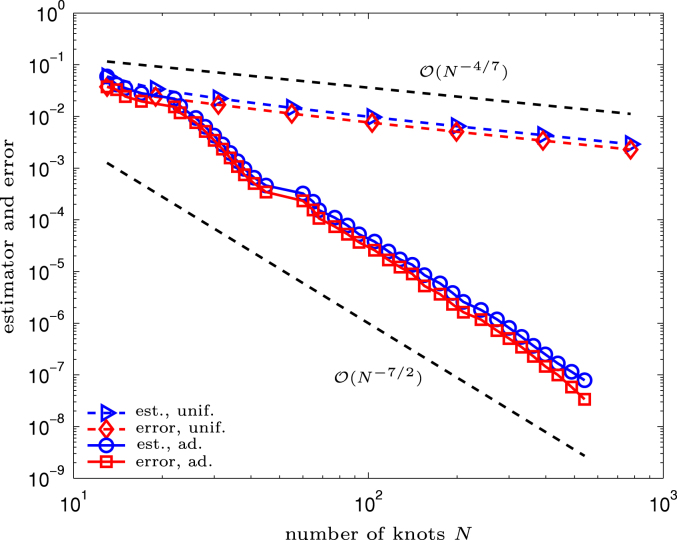
Experiment with singular solution on pacman geometry from Section  5.3. Error and estimator are plotted versus the number of knots  N.

In Fig. 5.4, error and error estimator are plotted. As the respective curves are parallel, we empirically confirm efficiency and reliability of the Faermann estimator. For the calculation of the error, we used $\left|\;\right|\;\left|\phi \right|\;{\left|\;\right|}^{2}=0.083525924784082$ in (5.12) which is obtained by Aitken’s $\Delta^{2}$-extrapolation. Since the solution lacks regularity, uniform refinement leads to the suboptimal rate $O\left(N^{-4/7}\right)$, whereas adaptive refinement leads to the optimal rate  $O\left(N^{-7/2}\right)$.

**Fig. 5.5:**
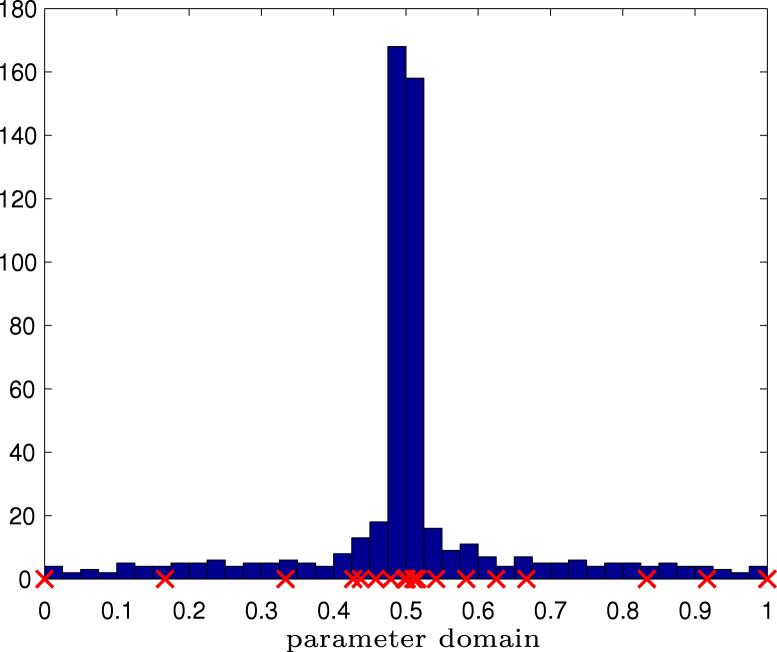
Experiment with singular solution on pacman geometry from Section  5.3. Histogram of number of knots over the parameter domain. Knots with maximal multiplicity p+1=3 are marked.

For adaptive refinement, Fig. 5.5 provides a histogram of the knots in [a,b] of the last refinement step. We see that the algorithm mainly refines the mesh around the singularity at t=1/2. Moreover, the multiplicity at the jump points t=1/3 and t=2/3 appears to be maximal so that the discrete solution $\phi_{\ell }$ also mimics the discontinuities of the exact solution ϕ. Hence the functions of the considered ansatz space do not need to be continuous there, see Theorem 4.1.

### 5.4 Adaptive IGABEM for slit problem

As last example, we consider a crack problem on the slit Γ=[−1,1]×{0}. For f(x,0)≔−x/2 and the single-layer operator V from (5.10), the exact solution of (1.1) reads

$$\phi \left(x,0\right)=\frac{-x}{\sqrt{1-x^{2}}}\text{.}$$

Note that $\phi \in {\overset{˜}{H}}^{-\varepsilon }\left(\Gamma \right)\setminus L^{2}\left(\Gamma \right)$ for all ε>0 and that ϕ has singularities at the tips x=±1. We parametrize Γ by the NURBS curve induced by

p=1,

$${\overset{ˇ}{K}}_{0}=\left(0,0,\frac{1}{5},\frac{2}{5},\frac{3}{5},\frac{4}{5},1,1\right)\text{,}$$

$$W_{0}=\left(1,1,1,1,1,1\right)\text{,}$$

$${\left(C_{i}\right)}_{i=1}^{N_{0}-p}=\left(\left(\begin{array}{c} -1\\ 0 \end{array}\right),\left(\begin{array}{c} -3/5\\ 0 \end{array}\right),\left(\begin{array}{c} -1/5\\ 0 \end{array}\right),\left(\begin{array}{c} 1/5\\ 0 \end{array}\right),\left(\begin{array}{c} 3/5\\ 0 \end{array}\right),\left(\begin{array}{c} 1\\ 0 \end{array}\right)\right)\text{.}$$

**Fig. 5.6:**
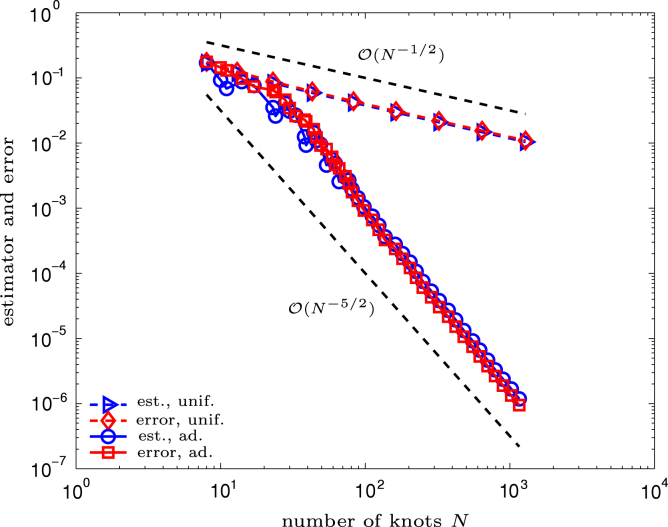
Experiment with singular solution on slit from Section  5.4. Error and estimator are plotted versus the number of knots  N.

In Fig. 5.6, error and error estimator for the uniform and for the adaptive approach are plotted. The error is obtained via (5.12), where $\left|\;\right|\;\left|\phi \right|\;{\left|\;\right|}^{2}=\pi /4$ is computed analytically. Since the solution lacks regularity, uniform refinement leads to the suboptimal rate $O\left(N^{-1/2}\right)$, whereas adaptive refinement leads to the optimal rate $O\left(N^{-5/2}\right)$.

**Fig. 5.7:**
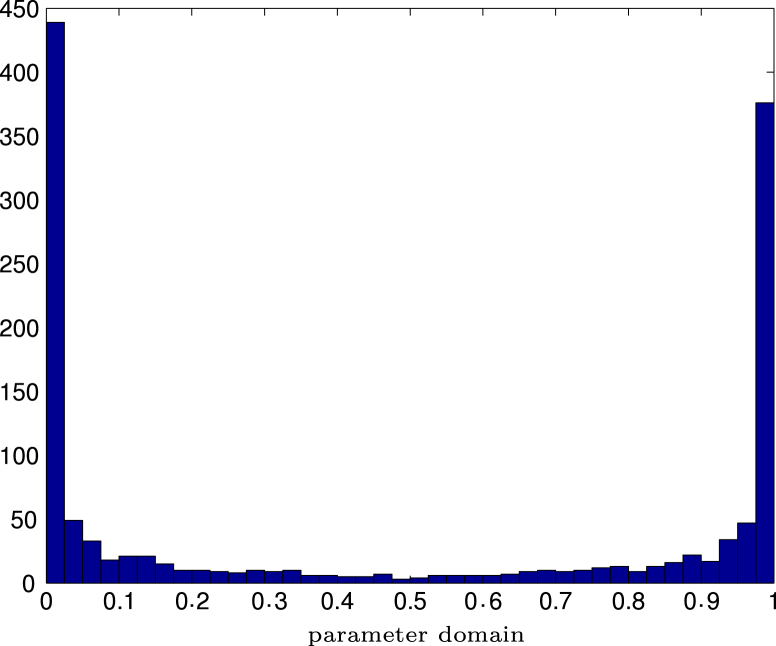
Experiment with singular solution on slit from Section  5.4. Histogram of number of knots over the parameter domain.

For adaptive refinement, we plot in Fig. 5.7 a histogram of the knots in [a,b]=[0,1] of the last refinement step. As expected, the algorithm mainly refines the mesh at the tips t=0 and t=1.

## 6 Conclusion

In this paper, we studied the residual-based a posteriori error estimator proposed by Faermann  [17] for the Galerkin boundary element method (BEM) in 2D. We proved reliability as well as efficiency of this estimator, where reliability requires that the ansatz space is sufficiently rich. This property is in particular satisfied for NURBS (see Theorem 4.4), and thus our result provides a first a posteriori error estimator for isogeometric BEM (IGABEM). Based on this error estimator, we formulated an adaptive algorithm for IGABEM, which uses h-refinement as well as multiplicity increase of the knots for refinement.

### 6.1 Analytical results

The numerical analysis of the pioneering work  [17] is restricted to the case of transformed spline ansatz spaces, where the arclength parametrization is used to define polynomial ansatz spaces on the boundary. It is our contribution, first, to work out the essential properties of the BEM spaces, which are needed to prove reliability and efficiency (see (A1)–(A2)), and, second, to show their validity in the case of IGABEM. Additionally, we propose a mesh-refining adaptive algorithm, which, in contrast to the one proposed in  [17] and the state-of-the-art concepts  [25], also steers the continuity properties of the discrete solution at the knots.

### 6.2 Numerical results

We considered the weakly-singular integral equation associated to the 2D Laplace problem. The conclusions of the three examples in Sections 5.2–5.4 are analogous and can be described as follows: Compared to uniform refinement, we always observed a superior convergence rate (or at least an equal one in the case of a smooth solution) of our proposed adaptive strategy. Indeed, the convergence rates of adaptive refinement are optimal with respect to the number of knots. Our algorithm is capable to detect the singularities of the (in general unknown) solution and to mainly perform refinement at these points. Moreover, jumps of the solution are automatically detected and the discontinuity is adaptively included in the ansatz spaces.

### 6.3 Open questions and future work

As already mentioned, all considered numerical experiments show optimal convergence of the estimator and the error. Thus, it is a goal of our future research to understand this observation mathematically in the spirit of  [25]. However, it is questionable if an analogous version of the reduction property on refined element domains  [25, (A2)], can be proved for the Faermann estimator $\eta_{h}$. Indeed, this is yet an open problem even for standard BEM with piecewise polynomials; see  [26], where at least convergence of an h-adaptive algorithm is analyzed. For the weighted-residual error estimator $\mu_{h}≔{\left\|h^{1/2}{\left(f-V\Phi_{h}\right)}^{\prime }\right\|}_{L^{2}\left(\Gamma \right)}$ proposed in  [16], the axioms of  [25] are satisfied for standard BEM with piecewise polynomials, see  [25, Section 5.4]. In  [22, Section 3.2], we show that the Faermann estimator can always be bounded from above by $\mu_{h}$, i.e.,  $\eta_{h}\leq C\mu_{h}$, where C>0 depends only on Γ. This especially implies reliability of $\mu_{h}$ for Galerkin IGABEM. Therefore, a natural goal is to prove optimal convergence of our adaptive algorithm for the estimator $\mu_{h}$.

Finally, the ultimate goal is of course to apply the estimators $\eta_{h}$ and $\mu_{h}$ in 3D Galerkin IGABEM. One then has to consider, e.g., T-splines  [5] or hierarchical B-splines  [27], since, in contrast to multivariate NURBS, they naturally allow for local mesh refinement. For standard BEM with piecewise polynomials,  [18] shows reliability and efficiency for $\eta_{h}$, whereas  [16] proves reliability. [25, Section 5.4] proves optimal convergence of adaptive h-refinement for $\mu_{h}$, while the estimate $\eta_{h}≲\mu_{h}$ as well as plain convergence for $\eta_{h}$-based adaptivity is analyzed in  [26]. The transfer of the mentioned results from standard BEM to adaptive IGABEM leaves interesting and challenging questions for future research.
